# YebC regulates variable surface antigen VlsE expression and is required for host immune evasion in *Borrelia burgdorferi*

**DOI:** 10.1371/journal.ppat.1008953

**Published:** 2020-10-13

**Authors:** Yan Zhang, Tong Chen, Sajith Raghunandanan, Xuwu Xiang, Jing Yang, Qiang Liu, Diane G. Edmondson, Steven J. Norris, X. Frank Yang, Yongliang Lou

**Affiliations:** 1 Wenzhou Key Laboratory of Sanitary Microbiology, Key Laboratory of Laboratory Medicine, Ministry of Education, School of Laboratory Medicine and Life Sciences, Wenzhou Medical University, Wenzhou, China; 2 Optometry and Eye Hospital and School of Ophthalmology, School of Biomedical Engineering, Wenzhou Medical University, Wenzhou, China; 3 Department of Microbiology and Immunology, Indiana University School of Medicine, Indianapolis, Indiana, United States of America; 4 Department of Pediatrics, Division of Medical Genetics, Duke University, Durham, North Carolina, United States of America; 5 Department of Anesthesiology, The First Affiliated Hospital, College of Medicine, Zhejiang University, Hangzhou, China; 6 Department of Pathology and Laboratory Medicine, UTHealth Medical School, Houston, Texas, United States of America; University of Montana, UNITED STATES

## Abstract

*Borrelia burgdorferi*, the Lyme disease pathogen causes persistent infection by evading the host immune response. Differential expression of the surface-exposed lipoprotein VlsE that undergoes antigenic variation is a key immune evasion strategy employed by *B*. *burgdorferi*. Most studies focused on the mechanism of VlsE antigen variation, but little is known about VlsE regulation and factor(s) that regulates differential *vlsE* expression. In this study, we investigated BB0025, a putative YebC family transcriptional regulator (and hence designated BB0025 as YebC of *B*. *burgdorferi* herein). We constructed *yebC* mutant and complemented strain in an infectious strain of *B*. *burgdorferi*. The *yebC* mutant could infect immunocompromised SCID mice but not immunocompetent mice, suggesting that YebC plays an important role in evading host adaptive immunity. RNA-seq analyses identified *vlsE* as one of the genes whose expression was most affected by YebC. Quantitative RT-PCR and Western blot analyses confirmed that *vlsE* expression was dependent on YebC. *In vitro*, YebC and VlsE were co-regulated in response to growth temperature. In mice, both *yebC* and *vlsE* were inversely expressed with *ospC* in response to the host adaptive immune response. Furthermore, EMSA proved that YebC directly binds to the *vlsE* promoter, suggesting a direct transcriptional control. These data demonstrate that YebC is a new regulator that modulates expression of *vlsE* and other genes important for spirochetal infection and immune evasion in the mammalian host.

## Author summary

The Lyme disease pathogen evolves strategies to subvert host immune responses to cause persistent infection. Antigen variation is a common strategy employed by some pathogens to escape immune recognition and clearance. The VlsE antigen variation system, discovered in 1997, is one such mechanism by which *Borrelia burgdorferi*, evades host immune responses. *vlsE* expression increases concomitantly with downregulation of the major immunodominant surface lipoprotein OspC in response to host adaptive immune response activation. But factors that regulates the differential expression of *vlsE* have not been well studied. In present study, we identified a key transcription factor, YebC (BB0025), that regulates *vlsE* expression. A mutant lacking YebC has decreased levels of VlsE and was attenuated in immunocompetent mice but was virulent in immunocompromised mice. Identification of YebC as a regulator for *vlsE* sets the foundation to further study how the pathogen senses the immune pressure to activate the VlsE antigenic variation system and provides a potential therapeutic target to combat persistent Lyme disease.

## Introduction

Lyme disease is the most commonly reported arthropod-borne infection in the United States and Europe, and is also found in Asia [1]. The causative agents of Lyme disease are members of the genus *Borrelia* (including *B*. *burgdorferi*, *B*. *garinii*, and *B*. *afzelii*), which are transmitted to mammals via the bite of *Ixodes* ticks. During the transmission and colonization in both tick vectors and mammalian hosts, *B*. *burgdorferi* dramatically regulates its gene expression [2–6]. In the past two decades, several key regulators/pathways have been identified that govern differential expression of *B*. *burgdorferi* during the tick-mammal transmission. These include two sets of two-component systems, with each modulating the adaptation to each of the two hosts [5]. Hk1/Rrp1, a c-di-GMP producing system, controls spirochete’s adaptation to the tick vector [7–13], whereas Hk2/Rrp2 is essential for *B*. *burgdorferi* to establish infection in the mammalian host [14–17]. Rrp2, along with transcriptional activator BosR and repressor BadR, activates the RpoN-RpoS (σ^54^-σ^S^) sigma cascade, which in turn controls the production of OspC and several virulence factors [14–28]. Several additional regulators have been identified that differentially regulate gene expression during the tick-mammal transmission including DsrA [29], Hfq [30], Hbb [31,32], CsrA [33–35], BpuR [36], EbfC [37], BpaB [38], SpoVG [39], BBD18 [40], LptA [41], BadP [42] and DksA [43,44].

Differential gene expression of *B*. *burgdorferi* is vital to its transition from the early phase to the persistent phase of mammalian infection. In the first week of infection, expression of *ospC*, which is essential for the early stage of spirochetal infection, is high [45–47]. As a highly immunogenic surface lipoprotein, OspC levels become downregulated as the host adaptative immune response is activated [48, 49]. Meanwhile, another surface-exposed lipoprotein, VlsE, which is structurally similar to OspC but antigenically variable, is upregulated [47,49,50]. Antigen variation of VlsE is achieved via gene conversion that occurs between the expressing *vlsE* locus and the adjacent 15 silent cassettes, each of which contains six VlsE antigenic variable regions [50–52]. Spirochetes lacking *vlsE* are able to maintain persistent infection in immunocompromised mice, but are unable to sustain infection in immunocompetent mice [53–57]. Despite the importance of VlsE in immune evasion, very little is known about the mechanism of differential expression of *vlsE* [51,52].

**Fig 1:**
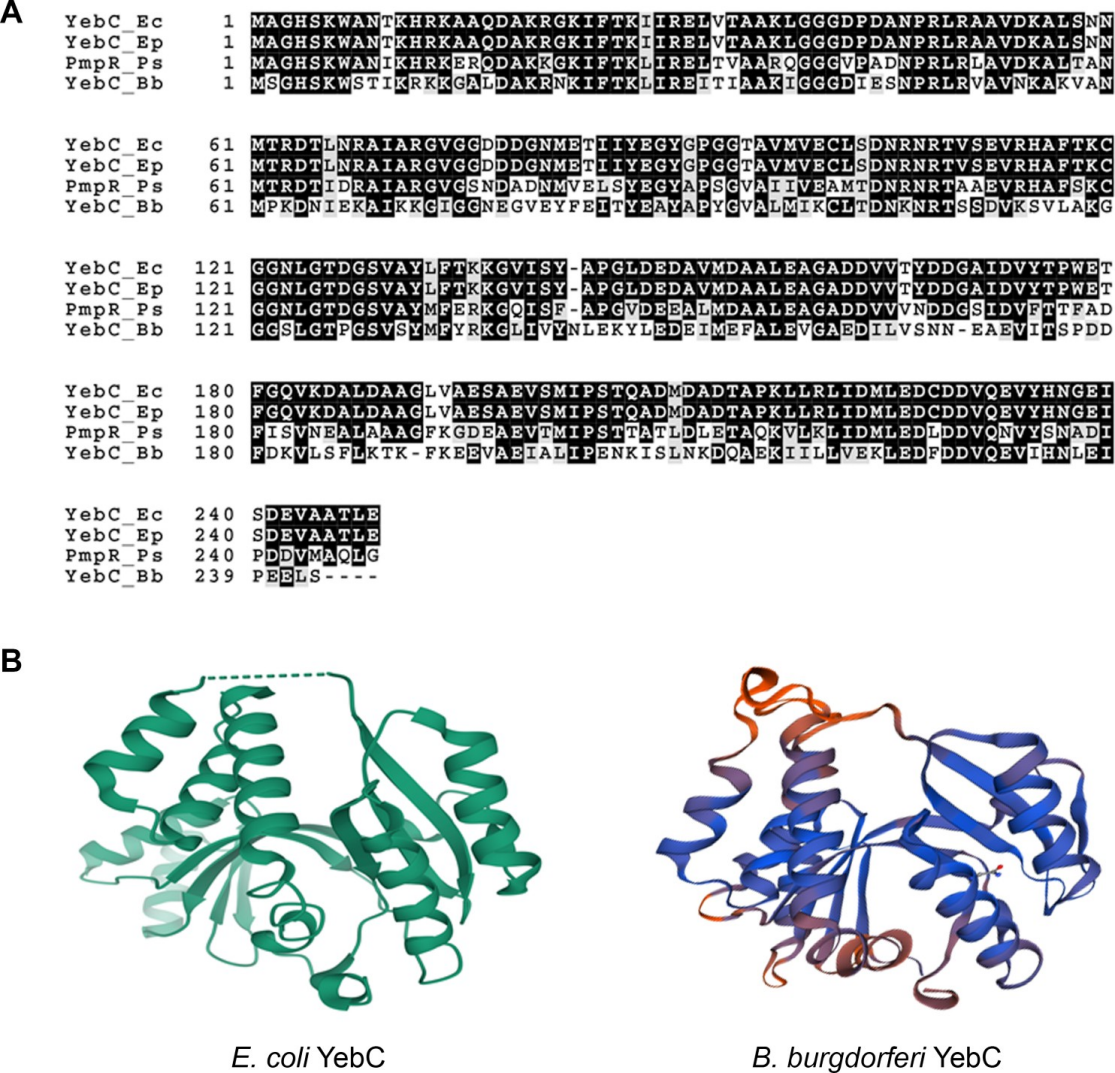
Alignment of YebC of *B*. *burgdorferi* (BB0025) with other members of YebC/PmpR family transcriptional regulators. (**A**), YebC of *B*. *burgdorferi* was aligned with three other YebC/RmpR family proteins (TACO1 family, pfam PF01709), YebC from *E*. *coli* (Ec), PmpR_Ps, PmpR from *P*. *aeruginosa* and YebC from *Edwardsiella piscicida* (Ep). Multiple alignment was generated with CLUSTALW (https://www.genome.jp/tools-bin/clustalw) and the figure was draw with BoxShade (https://embnet.vital-it.ch/software/BOX_form.html). (**B**), Homology model for *B*. *burgdorferi* YebC. The model was generated using the crystal structure of *E*. *coli* YebC (1KON) as template and SWISS-MODEL program (https://swissmodel.expasy.org/interactive/HvXRyP).

*B*. *burgdorferi* has a reduced genome with relatively few known or predicted transcriptional regulators [58]. BB0025, originally assigned as a hypothetical protein, recently was annotated as a putative YebC/PmpR family DNA-binding transcriptional regulator (TACO1 family, pfam PF01709) in the UniProt database (**Fig 1**). Herein, we designated BB0025 as YebC of *B*. *burgdorferi*. Although functions of this group of proteins are largely unknown, several studies suggest that they play important roles in gene regulation and pathogenesis. In *Pseudomonas aeruginosa*, PmpR is involved in regulation of the quinolone signal (PQS) system and of pyocyanin production [59]. Disruption of *yebC* in *Escherichia coli* resulted in reduced survival upon on exposure to extreme ionizing radiations [60]. In *Lactobacillus*, YebC was shown to regulate proteolytic activity by binding to the promoter region of genes involved in proteolysis [61]. More recently, YebC of *Edwardsiella piscicida* was shown to control virulence by directly binding to the promoter and activating its Type III secretion system [62]. In current study, we discovered that YebC of *B*. *burgdorferi* is a new regulator that controls expression of *vlsE* and other genes important for mammalian infection of *B*. *burgdorferi*.

## Results

### Construction of a *yebC* mutant and the complemented strain

**Fig 2:**
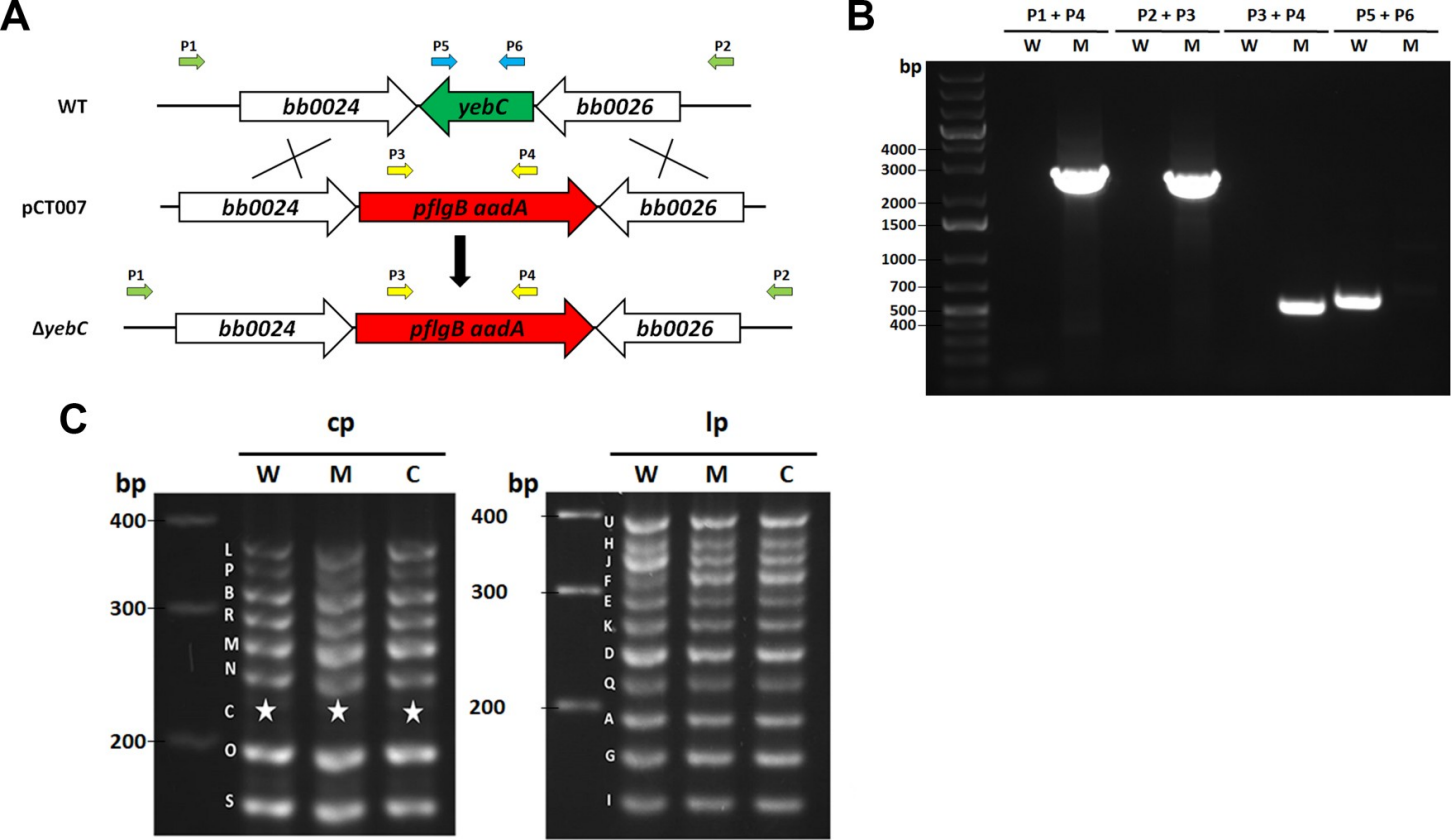
Construction of the *yebC* mutant and the complemented strain. **(A)** Strategy for constructing the *yebC* mutant. WT: genomic context of *yebC* in the parent strain, 5A4NP1 (referred to here as WT). pCT007: the suicide vector used for inactivation of *yebC*. Δ*yebC*: the *yebC* mutant. Arrows indicate the primers used for PCR analyses. **(B)** PCR analyses of the wild-type (W) and the *yebC* mutant (M) strains. The specific primer pairs used are indicated at the top. **(C)** Endogenous plasmid profiles of each strain by multiplex PCR analyses as previously described [82]. cp, circular plasmid; lp, linear plasmid. Letters on the left indicate the bands corresponding to each endogenous plasmid that was defined previously for the *B*. *burgdorferi* strain B31genome [58, 84]. ★ indicates the band corresponding to plasmid C (cp9) that is missing in all three strains.

**Fig 3:**
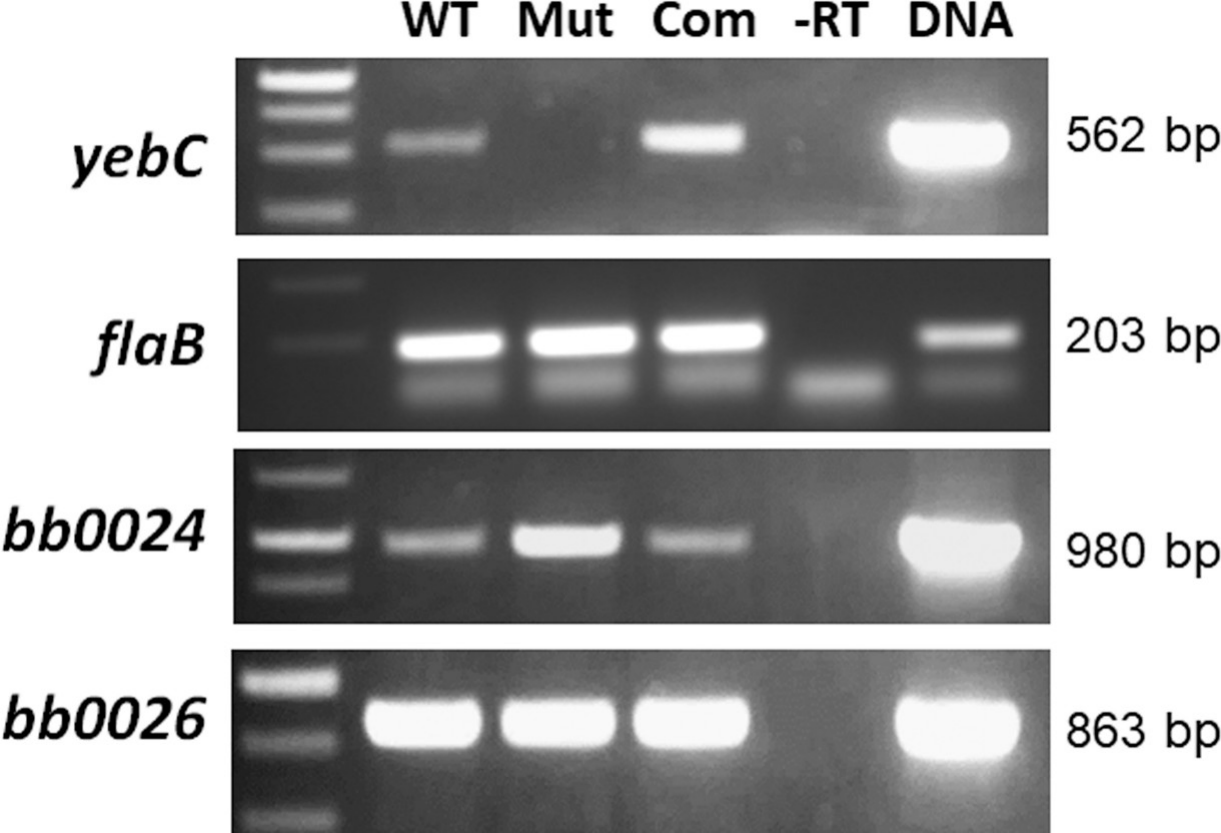
RT-PCR analyses of expressions of *yebC*, *bb0024* and *bb0026*. Wild-type 5A4NP1 (WT), the *yebC* mutant (Mut) and the complemented strain (Com) were cultured in BSK-II medium, and then were collected at the mid-log phase (~3×10^7^ spirochetes/ml). RNA was extracted and subjected to RT-PCR analyses. *flaB* serves as an internal control. “-RT”, RT-PCR reaction without of reverse transcriptase. “DNA”: PCR control using the genomic DNA as a template.

Sequence analyses revealed the BB0025 belongs to YebC/RmpR transcriptional regulator family (**Fig 1A**). Structural modeling of *B*. *burgdorferi* YebC based on the crystal structure of *E*.*coli* YebC demonstrated strong structural similarity to *E*.*coli* YebC (**Fig 1B**). To investigate the function of YebC, we constructed a *yebC* mutant by allelic exchange in the low-passage, infectious strain of *B*. *burgdorferi* strain B31 clone 5A4NP1 [63]. A suicide vector pCT007 was constructed with an *aadA* gene (which confers streptomycin-resistance) with flanking upstream and downstream regions of *yebC* (**Fig 2A**), and transformed into 5A4NP1. Streptomycin-resistant *Borrelia* transformants were analyzed by PCR for confirming *yebC* deletion (**Fig 2B**). One of the *yebC* mutant clones that had plasmid profiles identical to that of 5A4NP1 was chosen for complementation with pCT016, a pBSV2-derived shuttle vector with a gentamicin-resistance cassette carrying a wild-type copy of *yebC* with its native promoter (**Fig 2C**). A complemented clone that had an identical endogenous plasmid profiles to that of the *yebC* mutant was selected for further study (**Fig 2C**). The RT-PCR result confirmed that the *yebC* mutant no longer expressed *yebC*, and the complemented strain restored *yebC* expression (**Fig 3**). In addition, RT-PCR confirmed that deletion of *yebC* did not affect expression of the adjacent genes *bb0024* and *bb0026* (**Fig 3**).

### The *yebC* mutant was not able to infect immunocompetent mice

During *in vitro* cultivation, the *yebC* mutant did not show any significant difference in growth when compared to parental strain. To examine the *yebC* mutant’s phenotype *in vivo*, groups of C3H/HeN mice were needle inoculated with the 5A4NP1 parent strain (hereafter referred to as wild-type strain), the *yebC* mutant, or the complemented strain. Ear punch biopsies were collected at 2, 3, 4 weeks post-infection and cultured in BSK-II medium for the presence of spirochetes. At 4-weeks post-infection, all mice were sacrificed and several mouse tissues including ear, joint, heart, skin, and bladder were collected and cultured. As shown in **Table 1**, mice inoculated with the wild type or the complemented strain were culture-positive at each time point of post-infection with two different doses (1×10^5^ or 1×10^6^ spricochetes/mouse). No mice were infected with 1×10^5^ of the *yebC* mutant (**Table 1**). However, at high dose of infection (1×10^6^), after a prolonged culture of mouse tissues in BSK-II medium (> 2 weeks), two mice infected with the *yebC* mutant showed culture positive for some tissues, while remaining eight were culture negative (**Table 1**). These data indicate that the *yebC* mutant has a defect in establishing infection in immunocompetent mice.

**Table 1:** Infection of the *yebC* mutant in immunocompetent mice (C3H/HeN).

Strains and infection duration	No. cultures positive/No. tested					No. infected/Total No. of mice
	Ear	Joint	Heart	Skin	Bladder	
5A4NP1 (1 × 10^5^, 1 × 10^6^)						
2 weeks	5/5					5/5
3 weeks	5/5					5/5
4 weeks	5/5	5/5	5/5	5/5	5/5	5/5
△*yebC* (1 × 10^5^)						
2 weeks	0/5					0/5
3 weeks	0/5					0/5
4 weeks	0/5	0/5	0/5	0/5	0/5	0/5
△*yebC* (1 × 10^6^)						
2 weeks	0/10					0/10
3 weeks	0/10					0/10
4 weeks	0/10	0/10	1/10	2/10	2/10	2/10
*yebC*^com^ (1 × 10^5^, 1 × 10^6^)						
2 weeks	3/5					3/5
3 weeks	5/5					5/5
4 weeks	5/5	5/5	5/5	5/5	5/5	5/5

We further examined whether the *yebC* mutant could infect immunocompromised mice. Groups of SCID mice were needle inoculated with wild-type strain, the *yebC* mutant, or complemented strain with a dose of 1×10^6^ spirochetes per mouse. All mice infected with wild type or the complemented strain were culture positive at all time points of post infection (**Table 2**). No mice yielded positive cultures at 2 weeks post-infection with the *yebC* mutant, 3 out of 6 mice were culture positive at 3 weeks post infection, and all mice were culture positive at 4 weeks post infection. These observations suggest that the *yebC* mutant was able to establish infection in immunocompromised mice despite a delayed infectious course.

**Table 2:** Infection of the *yebC* mutant in immunodeficient mice (SCID).

Strains and infection duration*	No. cultures positive/No. tested					No. infected/Total No. of mice
	Ear	Joint	Heart	Skin	Bladder	
5A4NP1 (parent strain)						
2 weeks	6/6					6/6
3 weeks	6/6					6/6
4 weeks	6/6	6/6	6/6	6/6	6/6	6/6
△*yebC*						
2 weeks	0/6					0/6
3 weeks	3/6					3/6
4 weeks	6/6	6/6	6/6	6/6	6/6	6/6
*yebC*^com^						
2 weeks	3/3					3/3
3 weeks	3/3					3/3
4 weeks	3/3	3/3	3/3	3/3	3/3	3/3

* Dose of infection: 1 × 10^6^ spirochetes per mouse

### Transcriptome analyses of the *yebC* mutant

**Fig 4:**
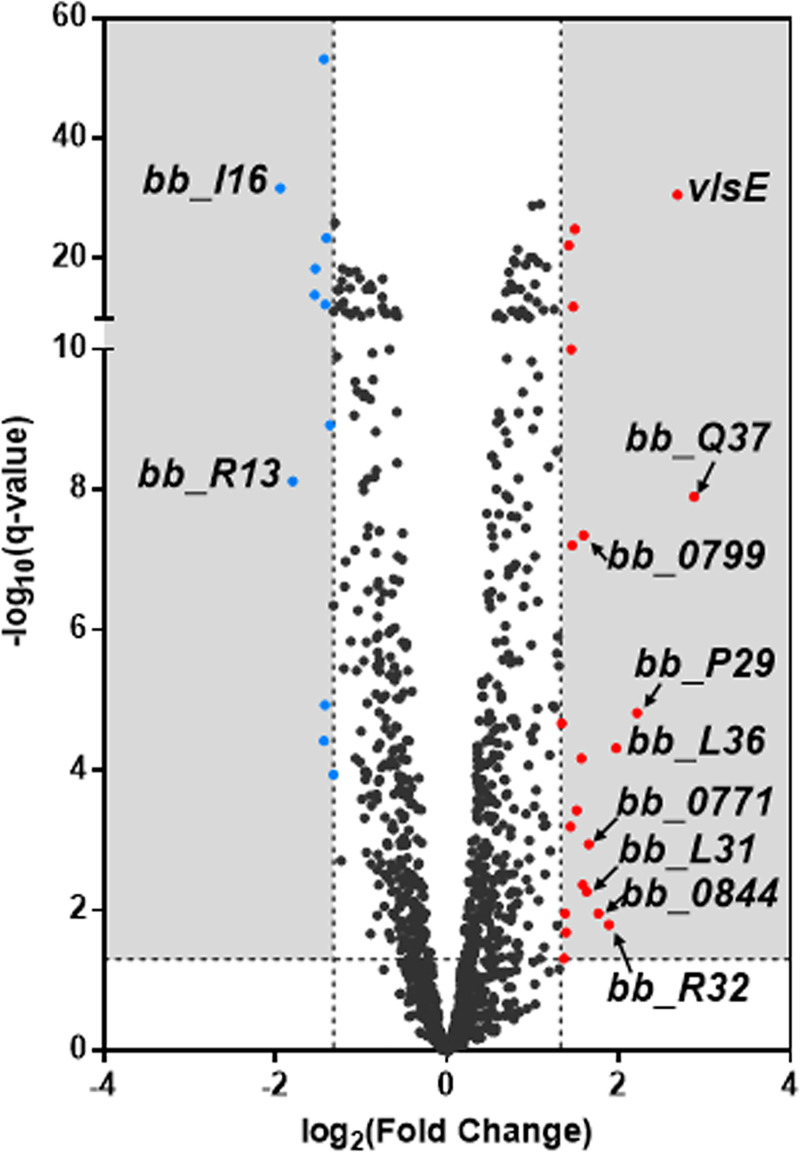
Volcano plots of differentially expressed genes. The volcano plot depicts log_2_ fold change on the *x*-axis and False Discovery Rate adjusted *p* value (q-value) on the *y*-axis. Single genes are depicted as dots. The transcript levels of 33 genes had greater than 2.5-fold change in the *yebC* mutant relative to the parental strain (p < 0.05). 22 genes were upregulated (in red) in the parent relative to the mutant, and 11 genes were downregulated (in blue). Genes with greater than 3-fold change are labeled with their gene IDs.

To investigate the molecular mechanisms underlying the requirement of YebC for mammalian infection, we sought to identify YebC-regulated genes by RNA sequencing analyses. Wild-type 5A4NP1 and the *yebC* mutant were cultivated in BSK-II at 37°C and harvested at the mid-logarithmic growth. Comparison of the transcriptomes of the wild type and *yebC* mutant revealed a total of 33 genes whose expressions were either up- or down-regulated by YebC (> 2.5-fold) under standard *in vitro* culture conditions. Among these, 21 genes were positively regulated by YebC (**Table 3**), whereas 11 genes were negatively regulated by YebC (**Table 4**). Most of differentially regulated genes are located on various endogenous plasmids, especially on the redundant cp32 circular plasmids. Genes having >2.5-fold changes of expression were highlighted in the volcano plot, and genes with > 3-fold changes were highlighted and labeled (**Fig 4**). One of the genes most positively regulated by YebC was *vlsE*, which showed 6.43-fold reduction in the *yebC* mutant.

**Table 3:** RNA-seq analysis identification of genes that are positively regulated by YebC.

Locus	Common Name	Location	WT/ΔyebC*
BB_Q37	hypothetical protein	lp56	7.36
**BB_F0041**	**outer surface protein VlsE1**	**lp28-1**	**6.43**
BB_P29	hypothetical protein	cp32-1	4.64
BB_L36	protein BppA	cp32-8	3.92
BB_R32	hypothetical protein	cp32-4	3.70
BB_0844	lipoprotein	chromosome	3.39
BB_0771	RNA polymerase sigma factor RpoS	chromosome	3.14
BB_L31	hypothetical protein	cp32-8	3.08
BB_0799	hypothetical protein	chromosome	3.01
BB_L32	PF-32 protein	cp32-8	2.99
BB_K07	lipoprotein	lp36	2.96
BB_N02	hypothetical protein	cp32-9	2.85
BB_L29	hypothetical protein	cp32-8	2.81
BB_0438	DNA polymerase III subunit beta	chromosome	2.77
BB_0800	transcription termination/antitermination protein NusA	chromosome	2.74
BB_0801	translation initiation factor IF-2	chromosome	2.72
BB_F01	hypothetical protein	lp28-1	2.71
BB_F20	BBF20	lp28-1	2.67
BB_L27	protein BdrP	cp32-8	2.61
BB_S34	hypothetical protein	cp32-3	2.59
BB_R33	plasmid partition protein, putative	cp32-4	2.57
BB_N03	hypothetical protein	cp32-9	2.52

*Fold changes of gene expression of the wild-type strain with respect to the *yebC* mutant (the genes with fold change > 2.50). q (adjusted p value) < 0.05.

**Table 4:** RNA-seq analysis identification of genes that are negatively regulated by YebC.

Locus	Common Name	Location	WT/ΔyebC*
BB_I16	repetitive antigen A, VraA	lp28-4	-6.17
BB_R13	hypothetical protein BB_R13	cp32-4	-3.85
BB_A51	hypothetical protein BB_A51	lp54	-3.47
BB_A49	hypothetical protein BB_A49	lp54	-2.91
BB_N14	hypothetical protein BB_N14	cp32-9	-2.90
BB_0603	integral outer membrane protein p66	chromosome	-2.70
BB_S17	hypothetical protein BB_S17	cp32-3	-2.70
BB_0240	glycerol uptake facilitator	chromosome	-2.68
BB_G02	hypothetical protein	lp28-2	-2.67
BB_t31	NA		-2.65
BB_0001	hypothetical protein BB_0001	chromosome	-2.57

*Fold changes of gene expression of the wild-type strain with respect to the *yebC* mutant (the genes with fold change <-2.50). q (adjusted p value) < 0.05.

### Verification of genes positively or negatively regulated by YebC

**Fig 5:**
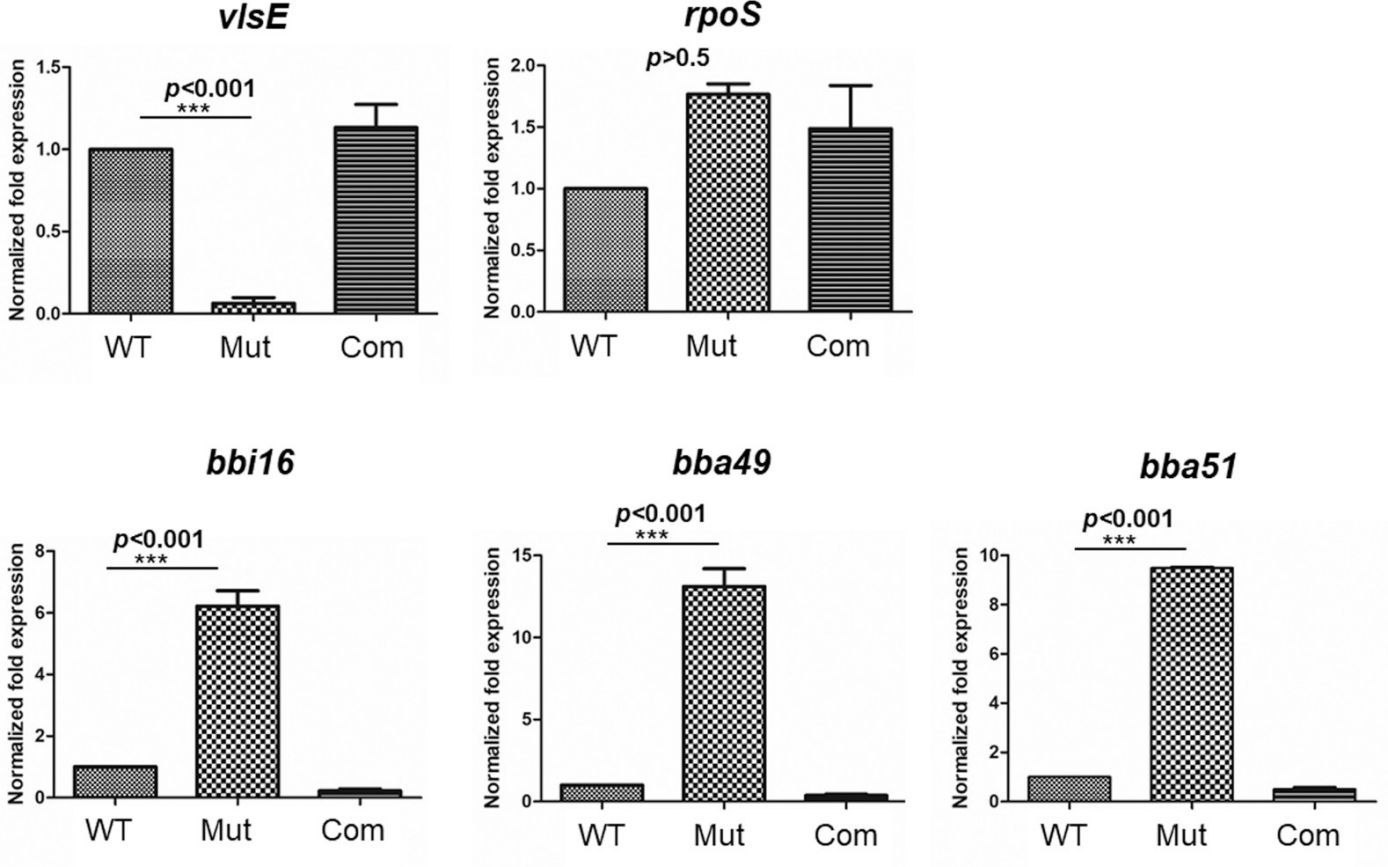
qRT-PCR verification of positively or negatively regulated genes by YebC identified by RNA-seq. Wild-type *B*. *burgdorferi* strain 5A4NP1 (WT), the *yebC* mutant (Mut) and the complemented strain (Com) were cultured in BSK-II medium at pH 7.5 and harvested at the mid-log phase. RNAs were extracted and subjected to qRT-PCR analyses for expression of *vlsE*, *rpoS*, *bbi16*, *bba49*, *bba51*. The expression levels for each gene in wild-type strain are set as 1.0. The bars represent the mean values of three independent experiments, and the error bars represent the standard deviation. ***, *p* < 0.001 using one-way Anova.

qRT-PCR analyses were performed to validate the expression of YebC-regulated genes identified by RNA-seq. Two positively regulated genes that are important for infection, *vlsE* and *rpoS* (*bb0771*), were examined. Consistent with RNA-seq results, qRT-PCR analyses also showed that *vlsE* expression was under the control of YebC (**Fig 5**). Even though *rpoS* showed 3.14-fold decrease of expression in the *yebC* mutant in RNA-seq, no significant difference was observed in qRT-PCR analysis. Three top negatively regulated genes, *bbi16*, *bba49*, and *bba51*, were assessed by qRT-PCR. The result confirmed that all the three genes were negatively regulated by YebC (**Fig 5**).

### YebC controls VlsE production *in vitro*

**Fig 6:**
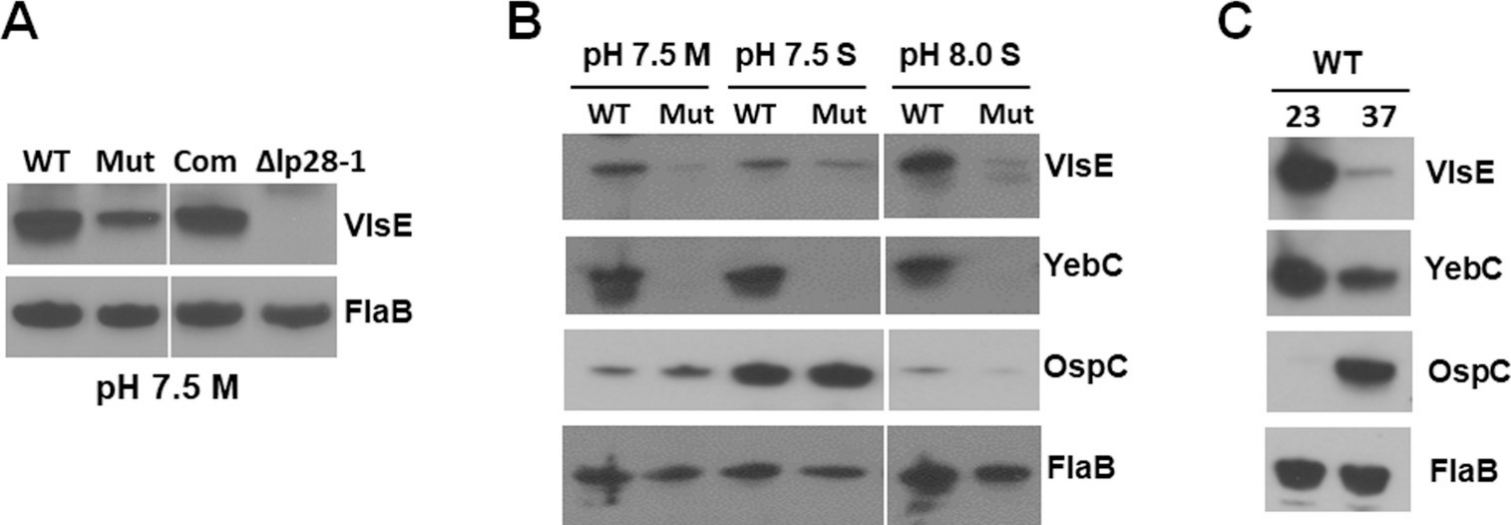
Immunoblot analysis of VlsE and YebC protein levels. Wild-type *B. burgdorferi* strain 5A4NP1 (WT), the *yebC* mutant (Mut), the complemented strain (Com), or a B31 clone lacking the entire plasmid lp28-1 (Δlp28-1) were cultured in BSK-II medium at pH 7.5, 37°C, and harvested at the mid-log phase (M) (**A**), or cultured under various pH conditions (pH 7.5, or 8.0) at 37°C, and harvested at either mid-log (M) or stationary phase (S) (**B**), or cultured at 23°C (pH 7.5) and harvested at stationary phase (**C**). Cell lysates were probed with antibodies against VlsE, YebC, OspC, or FlaB (loading control).

To determine whether YebC regulates VlsE protein levels, immunoblot analysis was performed with cells lysates from wild type, the *yebC* mutant, the complemented strain, and a *B*. *burgdorferi* strain lacking the entire plasmid lp28-1 that harbors *vlsE*. The result showed that the VlsE protein was dramatically reduced in the *yebC* mutant and levels were restored in the complemented strain. (**Fig 6A**). Previous reports showed that environmental cues can influence *vlsE* expression, although disparate results were observed [64–66]. We thus further investigated VlsE and YebC levels under various growth conditions. The result showed that cell density did not dramatically affect VlsE expression at the protein level, and elevated pH (pH 8.0) moderately increased VlsE production (**Fig 6B**). Consistent with previous reports, ambient temperature (23°C) dramatically increased the level of VlsE, which is inversely correlated with the level of OspC (**Fig 6C**) [64,65]. The level of YebC displayed a similar pattern of temperature-dependent regulation as that of VlsE, showing an increased production when grown at ambient temperature (**Fig 6C**).

### Correlative expression of *yebC* and *vlsE* in mice

**Fig 7:**
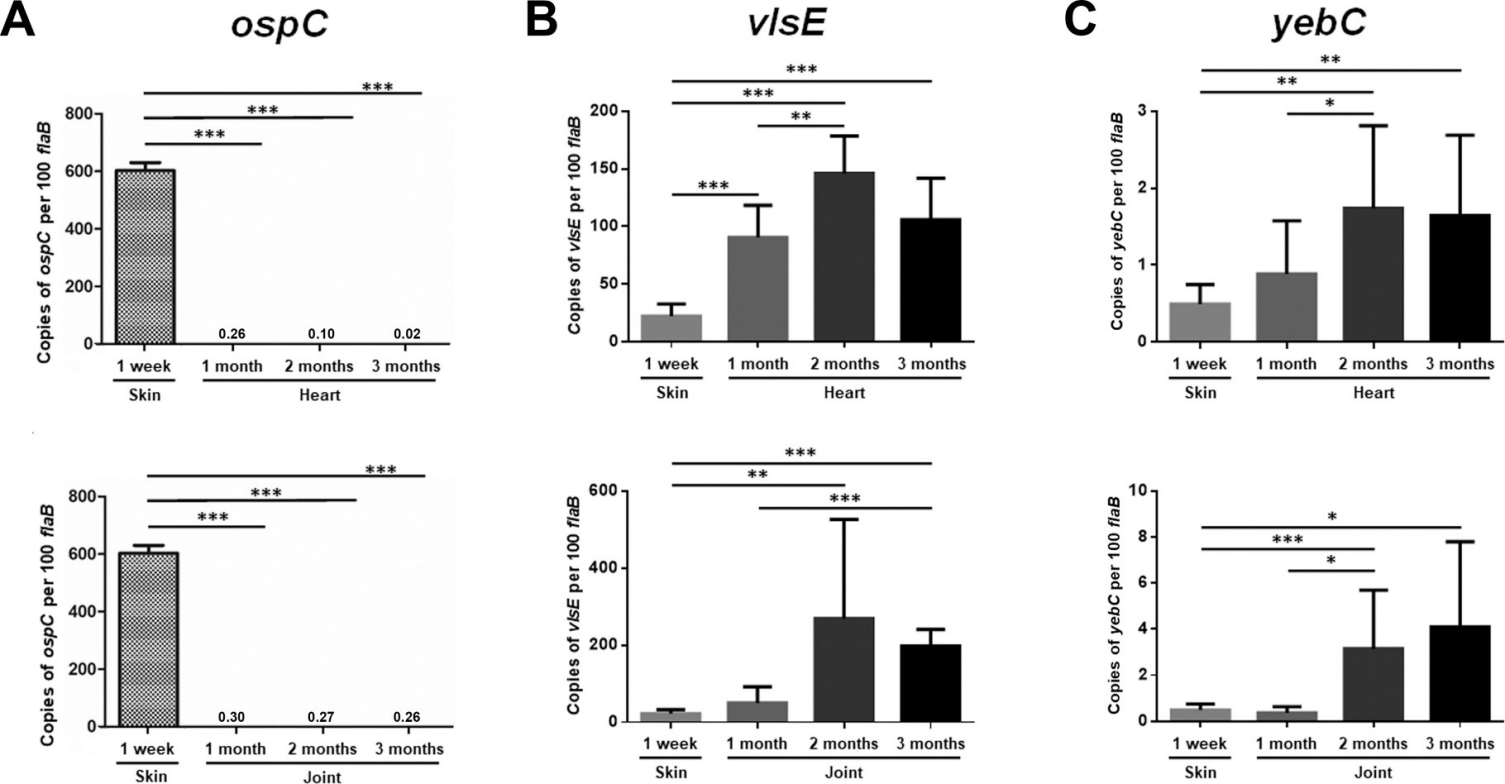
Analyses of *yebC*, v*lsE* and *ospC* expression in mouse tissues by qRT-PCR. Groups of C3H/HeN mice (n = 3 for each data point) were inoculated with 10^6^ of wild-type spirochetes (5A4NP1) via the intradermal dorsal route. Mice were euthanized at 1-week, and 1, 2, 3 months post infection and skin (site of infection), heart (top) and joint (bottom) tissues were collected and then were subjected to RNA extraction followed by qRT-PCR analyses. The *ospC* (**A**), *vlsE* (**B**), and *yebC* (**C**) transcripts were analyzed in mouse samples by qRT-PCR via absolute quantification. The values represent the average copy number of *ospC*, *vlsE or yebC* normalized relative to 100 copies of *flaB*. All data are collected from three independent experiments, and the bars represent the mean values, and the error bars represent the standard deviation. *, *p* < 0.05; **, *p* < 0.01; ***, *p* < 0.001.

*vlsE* is differentially expressed during mammalian infection: at the early stage of spirochetal infection, *vlsE* level is low whereas *ospC* is high. As the host adaptative immune response is activated, expression of *vlsE* increases when *ospC* becomes downregulated [45–50,53]. To gather further evidence that YebC modulates *vlsE* expression, *vlsE*, *ospC*, and *yebC* transcript levels were examined during the course of mammalian infection. Similar to what was reported previously, *ospC* expression was high at 1 week post-infection in skin at the site of inoculation, and then became undetectable in heart and joint tissues during persistent infection (1, 2, 3 months post infection) (**Fig 7A**). On the other hand, *vlsE* expression was low in skin at the site of inoculation at 1 week post-infection, and then dramatically increased (5 to 10-fold increase) at 1 to 3 months of post-infection (**Fig 7B**). The result showed that *yebC* displayed a similar pattern of differential expression as *vlsE* during infection (**Fig 7C**), supporting the hypothesis that YebC modulates *vlsE* expression during spirochete’ transition from early to persistent infection in mammals.

### YebC directly binds to the *vlsE* promoter

**Fig 8:**
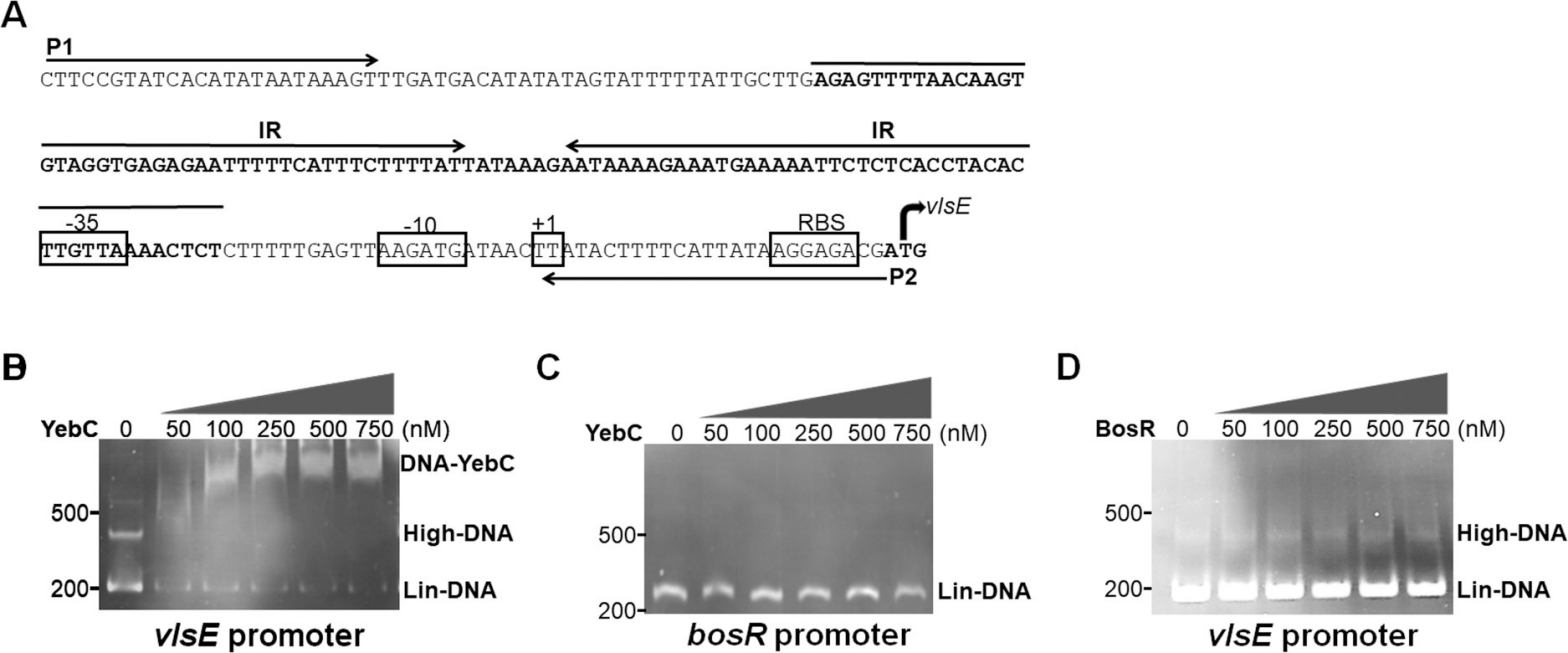
YebC binds to the *vlsE* promoter. **A.** Promoter sequence of the *vlsE* promoter. P1 and P2 are the primers used for PCR amplification of the DNA fragment for electrophoretic mobility shift analyses (EMSA). The inverted repeat sequences are labeled IR. -35 box of the *vlsE* promoter overlaps with the right arm of the IR. EMSA were performed using various concentrations of recombinant YebC (**B** and **C**) or BosR (**D**) proteins incubated with 50 nM of DNA fragments from 200 bp upstream of the *vlsE* promoter region or 300 bp upstream of the *bosR* promoter region (serving as a negative control). All the EMSA reactions include 0.01mg/ml BSA to prevent non-specific binding. Binding reactions were carried out for 30 min at 25°C, and then separated on a 5% polyacrylamide gel and stained with ethidium bromide. The band corresponding to the linear form DNA fragment (Lin-DNA), the higher form DNA (High-DNA), and the DNA-YebC protein complex (DNA-YebC), are labelled on the right.

Since YebC is predicted to be a transcriptional regulator, we postulate that YebC may directly bind to *vlsE* promoter to regulate its expression. Accordingly, recombinant YebC proteins were purified and electrophoretic mobility shift assays (EMSA) were performed using various concentrations of YebC proteins (ranging from 50 nM to 750 nM) and with 50 nM of 200-bp DNA fragment of the *vlsE* promoter region (**Fig 8A**). There is a 100-bp inverted repeat sequence overlapping the -35 region of the *vlsE* promoter in *B*. *burgdorferi* B31 [67] (**Fig 8A**). The inverted repeat DNA may form cruciform and higher form structures [68]. Interestingly, in the absence of YebC, two DNA bands were observed with the purified PCR DNA fragment from *vlsE* promoter under EMSA conditions (**Fig 8B**, first lane), but not with the control DNA fragment from the *bosR* promoter (**Fig 8C**, first lane). The lower band corresponded to the 200 bp linear form of the DNA fragment (Lin-DNA). We labeled the higher DNA band as High-DNA. Whether it is cruciform remains to be determined. A protein-DNA interaction was observed at 50 nM of protein concentration (1:1 of protein/DNA ratio) (**Fig 8B**). A larger protein-DNA complex was formed when protein concentrations increased (100 nM to 750 nM). No binding was evident when YebC protein was incubated with a DNA fragment from the *bosR* promoter (**Fig 8C**), nor when BosR protein incubated with the *vlsE* promoter (**Fig 8D**), indicating specific binding of YebC to *vlsE* promoter.

## Discussion

One of the unique features of *B*. *burgdorferi* infection is the ability of the spirochete to evade immune responses and maintain a persistent infection in the mammalian host [1,52]. Antigen variation and differential expression of VlsE plays a critical role in this process [46,50–52]. Over the past two decades, while much information regarding *Borrelia* gene regulation has been revealed, limited is known about regulation of *vlsE* expression [2,6,49,51,65–67]. In this study, we provide several lines of evidences to support the notion that YebC, a member of YebC/PmpR transcription factor, modulates differential expression of *vlsE*. First, genome-wide RNA-seq analysis showed that *vlsE* is one of the most regulated genes and its expression was dramatically reduced upon inactivation of *yebC* (**Fig 4**). Second, both *vlsE* and *yebC* expression were induced by ambient temperature when grown *in vitro* (**Fig 6**). YebC directly binds to the *vlsE* promoter (**Fig 7**). Third, the expression of *vlsE* and *yebC* correlated well during the course of infection in mice, consistent with the hypothesis that YebC regulates *vlsE* expression (**Fig 7**). Forth, YebC directly binds to the *vlsE* promoter (**Fig 8**). Lastly, the infection data showed that the *yebC* mutant could not infect immunocompetent mice efficiently but was still able to infect immunocompromised mice (**Tables 1** and **2**), indicating the essential role of *yebC* in evading the host adaptive immune system.

The YebC/PmpR family of proteins are widespread in all prokaryotes and eukaryotes [69]. Several recent structural and functional studies indicate that these proteins function as transcriptional regulators [69,70]. Bioinformatic analyses reveal that YebC family proteins can be divided into two subgroups [69]. Subgroup I *yebC* genes are adjacent to *ruvABC*, which encode for Holliday junction branch migrases and resolvase, suggesting that this subgroup of YebC proteins may be involved in regulating the process of Holliday junction resolution during DNA recombination. *B*. *burgdorferi* has a subgroup I YebC, and the *yebC* gene (*bb0025*) is adjacent to *ruvAB* (*bb0022-bb0023*). Interestingly, two groups independently reported that the mechanism underlying VlsE antigen variation is unique: unlike other bacteria, *B*. *burgdorferi* does not require RecA or other proteins generally involved in DNA recombination, repair, or replication, rather, it requires RuvAB for efficient *vlsE* recombination [71, 72]. Thus, along with the finding on YebC in present study, we postulate that the *ruvAB*-*yebC* gene cluster of *B*. *burgdorferi* plays a key role in modulating both processes of gene recombination and differential expression of *vlsE*.

Limited knowledge is known about the mechanism underlying differential expression of *vlsE*. Jutras et al. reported that SpoVG, a DNA-binding protein, binds to a sequence in the *vlsE* coding region of *B*. *burgdorferi* B31 [39]. As the binding sequence is located near the recombination site within the coding region of the *vlsE* gene, it may be involved in *vlsE* recombination rather than *vlsE* expression [39]. Drecktrah et. al, performed RNA-seq analysis and showed that RelBbu, a bifunctional synthetase/hydrolase (RelA/SpoT homolog) that is responsible to synthesize and hydrolyze the alarmones guanosine tetraphosphate and guanosine pentaphosphate [(p)ppGpp], upregulates *vlsE* expression. They also demonstrated that *vlsE* was upregulated upon starvation [66]. However, how RelBbu regulates vlsE expression remains to be elucidated. In this study, we showed that YebC directly binds to the *vlsE* promoter (**Fig 7B**). Hudson et al. first reported the presence of a long inverted repeat sequence overlapping with the -35 sequence of the promoter in *B*. *burgdorferi* B31 genome [67]. Recently, more IR sequences are reported in other *B*. *burgdorferi* strains [52]. The Function of this IR in *vlsE* expression remains unknown. These inverted repeats can form cruciform structures that have been recognized to play important biological roles [73]. Interestingly, we observed two bands of the *vlsE* promoter DNA under native conditions, and a single band on an agarose gel for the purified PCR product of the *vlsE* promoter fragment. Given that the lower band ran at 200-bp position and was the linear form of DNA, we postulate that the higher band (High-DNA) observed under the native condition could be cruciform DNA. Obviously, further direct structural evidence such as electron microscopy is needed to demonstrate that it is indeed the cruciform DNA. Nevertheless, one important observation is that both linear form and higher form of the DNA fragment were diminished when incubated with YebC (**Fig 7B**). We envision three scenarios based on this observation. 1, YebC is capable of binding to both linear form and cruciform of the *vlsE* promoter; 2, YebC can only bind to the linear form but stabilize it such that the equilibrium between linear and cruciform structures favors the linear form; 3, YebC can only bind to the cruciform structure and the linear form is eventually converted to cruciform. Given that YebC is a putative transcriptional activator that positively regulates *vlsE* expression, the second scenario makes sense: YebC binds and stabilizes the linear form of the *vlsE* promoter, drives conversion of cruciform to linear form, releases the -35 promoter region available for transcriptional activation. Further biochemical analysis is warranted to determine whether YebC has the activity to alter the thermodynamics of between linear and cruciform DNA structure.

Several factors, such as anti-OspC antibody, have been implied involving in regulation of *vlsE* expression [51]. The expression of *vlsE* increases as *B*. *burgdorferi* transitions from the early to persistent stages of mammalian infection, coinciding with the downregulation of *ospC* [47]. However, in SCID mice or B-cell-deficient mice, the transcript levels of both *vlsE* and *ospC* are high [47]. Furthermore, treatment of SCID mice with monoclonal antibodies against OspC resulted in dramatic decreases in *ospC* mRNA levels and increases in *vlsE* transcripts. These observations suggest that the induction of antibody responses against OspC may lead to the reciprocal regulation of *ospC* and *vlsE* observed in mice [47]. Co-culture of *B*. *burgdorferi* with endothelial cells was also shown to increase *vlsE* expression [67]. In addition, interferon γ expression was also reported to promote *vlsE* recombination, and possibly *vlsE* expression [74]. Whether *yebC* expression is in some manner linked to these host factors remain to be determined. In present study, RNA-seq analysis showed no influence of *yebC* deletion on *ospC* expression, suggesting that although YebC controls *vlsE* expression, it may not involve regulation of *ospC* under the *in vitro* culture conditions. Whether YebC regulates downregulation of *ospC in vivo* remains to be determined.

The role of VlsE of *B*. *burgdorferi* in evading host innate immune response was evidenced with the fact that a *B*. *burgdorferi* strain lacking the entire lp28-1 plasmid or an intact *vls* locus cannot sustain infection in wild-type mice at 2 week post-infection, but can maintain persistent infection in SCID mice [55–57,75,76]. The present study also showed that the *yebC* mutant had similar difference in infectivity in wild-type mice and SCID mice as that of the *vlsE* mutant (**Tables 1** and **2**), suggesting that an defect in *vlsE* expression is the key contributor to the *yebC* mutant’s infection phenotype. The *yebC* mutant could not establish infection in immunocompetent mice even at a dose of 1 X 10^5^ spirochetes/mouse, suggesting that YebC is essential for mammalian infection. We did observe that very few tissue samples became positive in mice inoculated with 1 X 10^6^ spirochetes/mouse, likely due to that fact that *yebC* deletion does not completely abolish *vlsE* expression and recombination during infection, as shown *in vitro* (**Fig 6**). The avirulent phenotype prevents us to examine *vlsE* expression in the *yebC* mutant in immunocompetent mice. The *yebC* mutant was infectious in SCID mice. Of note, the *yebC* mutant had delayed infection in SCID mice (**Table 2**). It is possible at lower dose of inoculation that the *yebC* mutant may show avirulent phenotype even in SCID mice since YebC may other functions in addition to VlsE. Regardless, because differential expression of *vlsE* and *ospC* does not occur in SCID mice, these mice are not suited for studying *vlsE* regulation. Therefore, we relied on the correlation of *yebC* and *vlsE* expression in wild-type spirochetes during the course of infection, which supports the hypothesis that YebC regulates *vlsE* expression (**Fig 7**).

It is reported that the *vlsE*-lacking spirochetes are capable of establishing infection in the early stage of infection in immunocompetent mice, and spirochetes can be detected in blood samples in first 1–2 weeks of infection [45–50,53]. One caveat of present study is that the infectivity of the *yebC* mutant was not examined at early time point of post-infection. We speculate that the *yebC* mutant may be deficient in establishing infection in mice even at this early time point, given that YebC regulates additional functions important for infection. In addition to *vlsE*, RNA-seq results showed that YebC regulates expression more than 33 genes (**Tables 3** and **4**). Many of these genes are located on the plasmids and have unknown functions. One gene whose function is known to be important for pathogenesis is *rpoS*, which showed over three-fold reduction in the *yebC* mutant. RpoS controls expression of *ospC*, *dbpBA* and several other genes important for mammalian infection [2,6,17,18]. However, this result could not be confirmed by qRT-PCR analysis (**Fig 4**). RNA-seq also did not show that the *yebC* deletion affected *ospC* and *dbpBA* expression. Thus, it is unlikely that YebC involves in regulation of the RpoS regulon. Other top positively regulated genes by YebC, including *bbq37*, *bbp29*, *bbl36* and *bbr32*, are members of paralogous families whose sequences are highly identical and could not be verified by qRT-PCR. Three top negatively regulated genes, *bbi16*, *bba49*, and *bba51*, were confirmed by qRT-PCR to be negatively regulated genes by YebC (**Fig 5**). *bbi16* is located on lp28-4, encoding VraA (virulent strain-associated repetitive antigen A), a surface antigen that shows partial protection with active immunization [77]. *bba49* and *bba51* are located on linear plasmid lp54, encoding conserved hypothetical proteins with unknown function [58]. Whether increased expressions of *bbi16*, *bba49* and *bba51* in the *yebC* mutant contributed to the avirulence phenotype remain to be determined.

Recently, Ramsey et al. used transposon insertion sequencing (Tn-seq) for a genome-wide screen and identified several factors important for resistance to NO, H_2_O_2_, and TBHP *in vitro* and YebC (BB0025) was identified as one of the genes that may be involved in H_2_O_2_ resistance [78]. Ramsey et al. also performed animal study with groups of pooled Tn mutants and found that *bb0025* Tn mutant was attenuated in both mouse strains. Our results using *yebC* deletion mutant was consistent with previous infection data using pooled Tn mutants. YebC’s role in defending host ROS may also explain why the *yebC* mutant had a delayed infection in SCID mice observed in this study (**Table 2**), as SCID mice have both macrophages and neutrophils that produce ROS. In addition, Ramsey et al., also showed that the *gp91phox*-/- mice lacking phagocyte superoxide production could not rescue the infectivity of the *bb0025* Tn mutant [78]. Given that YebC controls *vlsE* expression discovered herein, the phenotype of the *yebC* Tn mutant in the *gp91phox*-/- mice becomes evident.

In summary, this work identified YebC as the transcriptional factor that regulates *vlsE* gene expression, and it plays a vital role in mammalian infection of *B*. *burgdorferi*. This finding opens many exciting opportunities to study the mechanism of host immune evasion by *B*. *burgdorferi*. For example, how is YebC itself regulated, and what activates YebC during mammalian infection? How does IR influence YebC to regulate *vlsE* expression? Does YebC also modulate *vlsE* antigenic variation and contribute to the avirulent phenotype observed in the *yebC* mutant? In addition, given that YebC is also important for ROS response and that spirochetes encounter ROS during tick feeding in tick gut and salivary gland, it will be interesting to investigate whether YebC is important for the tick part of enzootic cycle of *B*. *burgdorferi*.

## Materials and methods

### Ethics statement

All animal experiments were approved by the IACUC committee of Indiana University School of Medicine under the protocol number # 11339. All experiments were in accordance with the institutional guidelines.

### *B*. *burgdorferi* strains and culture conditions

Low-passage, virulent *B*. *burgdorferi* strain 5A4NP1 (a gift from Drs. H. Kawabata and S. Norris, University of Texas Health Science Center at Houston) was used in this study. It was derived from wild-type *B*. *burgdorferi* strain B31 by inserting a kanamycin resistance marker in a restriction modification gene *bbe02* on plasmid lp25 [63]. Spirochetes were cultivated in Barbour-Stoenner-Kelly (BSK-II) medium supplemented with 6% normal rabbit serum (Pel-Freez Biologicals, Rogers, AR) [79] at 37°C with 5% CO_2_. At the time of growth, appropriate antibiotics were added to the cultures with the following final concentrations: 300 μg/ml for kanamycin, 50 μg/ml for streptomycin, and 50 μg/ml for gentamicin. The constructed suicide vector (pCT007) and shuttle vector (pCT016) were maintained in *Escherichia coli* strain DH5α. The antibiotic concentrations used for *E*. *coli* selection were as follows: kanamycin, 50 μg/ml; streptomycin, 50 μg/ml; and gentamicin, 10 μg/ml.

### Immunoblot analysis

Spirochetes from mid-log cultures were harvested by centrifuging at 8,000 × g for 10 min and followed by three time washing with PBS (pH 7.4) at 4°C. Pellets were suspended in SDS buffer containing 50 mM Tris-HCl (pH 8.0), 0.3% sodium dodecyl sulfate (SDS) and 10 mM dithiothreitol (DTT). Cell lysates (10^8^ cells per lane) were separated by 12% SDS-polyacrylamide gel electrophoresis (PAGE) and transferred to nitrocellulose membranes (GE-Healthcare, Milwaukee, WI). Membranes were blotted with mouse polyclonal antibody against VlsE (1:1,000 dilution) [76] and monoclonal antibody against FlaB (1:1,000 dilution) [41], and then incubated with goat anti-mouse lgG-HRP secondary antibody (1:1,000; Santa Cruz Biotechnology). Detection of horseradish peroxidase activity was determined by the enhanced chemiluminescence method (Thermo Pierce ECL Western Blotting Substrate) with subsequent exposure to X-ray film.

### Generation of the *yebC* mutant and the complemented strain

To inactivate the *yebC* in parent strain 5A4NP1, a suicide vector pCT007 was constructed for homologous recombination as following: the regions of DNA corresponding to 1.4 kb upstream and 1.4 kb downstream regions of *yebC* (**Fig 2A**) were PCR amplified from 5A4NP1 genomic DNA with primer pairs PRCT017/PRCT018 and PRCT019/PRCT020 (**Supplemental S1 Table**), respectively. The resulting PCR fragments were then cloned into upstream and downstream of an *aadA* streptomycin-resistant marker within the suicide vector pMP025 to generate the suicide vector pCT007. pCT007 was confirmed by both restriction enzyme digestion and sequencing, and then was transformed into 5A4NP1 as described previously [80,81]. Streptomycin-resistant *Borrelia* transformants were analyzed by PCR to confirm the correct *yebC* deletion (**Fig 2B**). Plasmid profiles of the confirmed *yebC* mutant clones were determined by multiplex PCR analyses with twenty pairs of primers specific for each of the endogenous plasmids as reported by Bunikis et al.[82]. One of the *yebC* mutant clones (BbCT006) that had plasmid profiles identical to those of 5A4NP1 was selected for complementation (**Fig 2C**). For trans complementation, a shuttle plasmid carrying a native promoter-driven *yebC* was generated as follows. The *yebC* regions flanked by *Bam*HI and *Pst*I restriction sites were amplified using primers PRCT092 and PRCT096 and then cloned into the shuttle vector pBSVG [83], resulting in in complementation plasmid named as pCT016. One complementation clone (BbCT009) that had an identical endogenous plasmid profiles to that of 5A4NP1 was selected for further study (**Fig 2C**).

### Mouse infection studies

Four-week-old C3H/HeN mice and C3H/SCID mice (Harlan, Indianapolis, IN) were subcutaneously inoculated with two doses of spirochetes (1×10^5^ and 1×10^6^) respectively. Ear punch biopsy samples were taken at 2 and 3 weeks post-injection. At 4 weeks post-injection, mice were euthanized, and multiple tissues (i.e. ear, joint, heart, skin and bladder tissues from each mouse) were harvested. All tissues were cultivated in 2 ml of the BSK-II medium (Sigma-Aldrich, St. Louis, MO) containing an antibiotic mixture of phosphomycin (2 mg/ml), rifampin (5 mg/ml), and amphotericin B (250 mg/ml) (Sigma-Aldrich) to inhibit bacterial and fungal contamination. All cultures were maintained at 37°C and examined for the presence of spirochetes by dark-field microscopy beginning from 5 days after inoculation. A single growth-positive culture was used as the criterion to determine positive mouse infection.

### qRT-PCR analyses

For verification of differentially expressed genes by YebC, wild-type *B*. *burgdorferi* strain 5A4NP1 (WT), the *yebC* mutant (Mut) and the complemented strain (Com) were cultured in BSK-II medium at pH 7.5 and harvested at the mid-log phase. RNAs were extracted and subjected to qRT-PCR analyses for expression of *vlsE*, *rpoS*, *bbi16*, *bba49*, *bba51*. Primers used were listed in Supplemental **S1 Table**. For *yebC*, *vlsE* and *ospC* expression in infected mice, four-week-old C3H/HeN mice were injected with wild-type strain 5A4NP1 at a dose of 1×10^6^ spirochetes per mouse. Mice were euthanized at different time points as indicated and mouse tissues were harvested and homogenized using the FastPrep-24 (MP Biomedicals). Total RNA was isolated using the TRIzol reagent (Thermo Fisher Scientific) according to the manufacturer’s instructions. To reduce trace amounts of DNA contamination, samples were further digested with RNase-free DNaseI (Qiagen), purified using the RNeasy mini kit (Qiagen) and analyzed with NanoDrop Spectrophotometer (Thermo Fisher Scientific). DNA-free RNA was confirmed by PCR amplification for the *B*. *burgdorferi flaB* gene. cDNA was synthesized using the PrimeScript 1st strand cDNA Synthesis Kit (TaKaRa). Given the low levels of bacterial RNA in mouse tissues, the specific primers for each gene target were used for cDNA synthesis instead of random primers. To quantify the transcript levels of genes of interest, an absolute quantitation method was used to create a standard curve for the qPCR assay according to the manufacturer’s protocol (Strategene, La Jolla, CA). Briefly, the PCR product of the *flaB* gene served as a standard template. A series of tenfold dilutions (10^2^-10^7^copies/ml) of the standard template was prepared, and qPCR was performed to generate a standard curve by plotting the initial template quantity against the Ct values for the standards. The quantity of the targeted genes in the cDNA samples was calculated using their Ct values and the standard curve. The samples were assayed in triplicate using a ABI 7000 Sequence Detection System and PowerUp SYBR Green Master Mix (Applied Biosystems). The levels of the target gene transcript were reported as per 100 copies of *flaB*.

### RNA Sequencing (RNA-seq)

Wild-type 5A4NP1 and the *yebC* mutant were cultivated in BSK-II at 37°C and harvested at the mid-logarithmic growth. RNA samples were extracted using the RNeasy mini kit (Qiagen, Valencia, CA) according to the manufacturer's protocol. Three independent culture samples were used for each strain. Removal of contaminated genomic DNA in the RNA samples was performed using RNase-free DNase I (Promega), and then confirmed by PCR amplification for the *flaB* gene. The concentration and quality of total RNA samples were first assessed using Agilent 2100 Bioanalyzer. A RIN (RNA Integrity Number) of 5 or higher was required to pass the quality control. rRNA removal was performed for each RNA sample (2 μg/sample) using Ribo-Zero rRNA Removal Kit (Bacterial, Illumina), and the RNA samples were then subjected to dual-indexed strand-specific cDNA library synthesis using TruSeq Stranded Total RNA Library Prep Kit (Illumina). Synthesized libraries were assessed for the quantity and size distribution using Qubit and Agilent 2100 Bioanalyzer. Two hundred picomolar pooled libraries were utilized per flow cell for clustering amplification on cBot using HiSeq 3000/4000 PE Cluster Kit and sequenced with 2×75bp paired-end configuration on HiSeq4000 (Illumina) using HiSeq 3000/4000 PE SBS Kit. A Phred quality score (Q score) was used to measure the quality of sequencing. More than 90% of the sequencing reads reached Q30 (99.9% base call accuracy). The sequencing data were first assessed using FastQC (Babraham Bioinformatics, Cambridge, UK) for quality control. Then all sequenced libraries were mapped to the *Borrelia burgdorferi* B31 genome (NCBI, GCA_000008685.2) using STAR RNA-seq aligner with the following parameter: “—outSAMmapqUnique 60”. The reads distribution across the genome was assessed using bamUtils software (from ngsutils). Uniquely mapped sequencing reads were assigned to *Borrelia burgdorferi* B31 refSeq genes using featureCounts (from subread) with the following parameters: “-s 2 -p–Q 10”. Quality control of sequencing and mapping results was summarized using MultiQC. Genes with read count per million (CPM) > 0.4 in more than 4 of the samples were kept. The data was normalized using TMM (trimmed mean of M values) method. Differential expression analysis was performed using edgeR. False discovery rate (FDR) was computed from *p* values using the Benjamini-Hochberg procedure. Genes with fold changes greater than 2.5 are listed in **Tables 1** and **2**. All genes are listed in supplemental file **S1 Data.** The RNA-seq data set was plotted on a volcano plot with the negative log of the *q* value on the y axis and the log_2_ of the fold change between wild type and the *yebC* mutant on the x axis using GraphPad Prism software (**Fig 4**).

### Electrophoretic mobility shift analyses (EMSA)

200-bp upstream to *vlsE* ORF and 350-bp region upstream to *bosR* were PCR amplified using specific sets of primers as listed in Supplementary **Table 1**. All the EMSA reactions were carried out in a 20 μL reaction mixture containing PCR amplified promoter DNA (50 nM), and various concentrations of recombinant YebC or BosR proteins (50 nM to 750 nM). Final 1 X EMSA buffer used for the reaction contained 10 mM Tris (pH 7.5), 1 mM EDTA, 100 mM KCl, 5% glycerol, 0.1 mM dithiothreitol and 0.01mg/ml BSA. Binding reactions were carried out for 30 min at 25°C. The reaction mix was then loaded on a 5% polyacrylamide gel and electrophoresis was carried out at 35 V at 4°C and later stained using ethidium bromide. Image was captured using a DigiDoc- Imaging System by UVP.

### Protein modeling

For molecular modeling of *B*. *burgdorferi* YebC, online tools Iterative Threading Assessment Server9 and SWISS MODEL were used. The pair-wise sequence alignment threshold was set to 70% by default. The template for generating model, *E*. *coli* YebC (PDB ID: 1KON) was taken from RCSB PDB (https://www.rcsb.org/). Further validation of the model was carried out employing RAMPAGE (http://mordred.bioc.cam.ac.uk/~rapper/rampage.php) and ERRAT (http://services.mbi.ucla.edu/ERRAT/). The model was further tested for all structural parameters which includes: MolProbity Score, 2.06; Clash score, 10.47; Ramachandran favored, 94.14%; Ramachandran outlayers, 1.46%; Rotamer outlayers, 1.46%; ERAT score, 7.

## Supporting information

S1 Table Strains, plasmids, and primers used in this study. (DOCX)

S1 Data Complete RNA-seq data. (XLSX)

## References

[1] Steere AC, Strle F, Wormser GP, Hu LT, Branda JA, Hovius JWR, et al (2016). Lyme borreliosis. Nat Rev Dis Primers. 2:16090 10.1038/nrdp.2016.90 27976670 PMC5539539

[2] Samuels DS (2011). Gene regulation in *Borrelia burgdorferi*. *Annu Rev Microbiol*. 65:479–99. 10.1146/annurev.micro.112408.134040 21801026

[3] Caimano MJ, Drecktrah D, Kung F, Samuels DS (2016). Interaction of the Lyme disease spirochete with its tick vector. Cellular microbiol. 18(7):919–27. 10.1111/cmi.12609 PMC5067140 27147446

[4] Stevenson B, Seshu J (2017). Regulation of Gene and Protein Expression in the Lyme Disease Spirochete *Curr Top Microbiol Immunol*. Berlin, Heidelberg: Springer Berlin Heidelberg p. 1–30. 10.1007/82_2017_49 29064060

[5] Ye M, Zhou Y, Lou Y, Yang XF (2016). Genome reduction of *Borrelia burgdorferi*: two TCS signaling pathways for two distinct host habitats. SCI CHINA LIFE SCI. 59(1):19 10.1007/s11427-015-4996-z 26740104 PMC5846617

[6] Radolf JD, Caimano MJ, Stevenson B, Hu LT (2012). Of ticks, mice and men: understanding the dual-host lifestyle of Lyme disease spirochaetes. Nat Rev Micro. 10(2):87–99. 10.1038/nrmicro2714 PMC3313462 22230951

[7] Sze CW, Smith A, Choi YH, Yang X, Pal U, Yu A (2013). Study of the response regulator Rrp1 reveals its regulatory role in chitobiose utilization and virulence of *Borrelia burgdorferi*. Infect Immun. 81(5):1775–87. 10.1128/IAI.00050-13 23478317 PMC3647990

[8] Sultan SZ, Pitzer JE, Boquoi T, Hobbs G, Miller MR, Motaleb MA (2011). Analysis of the HD-GYP domain cyclic dimeric GMP phosphodiesterase reveals a role in motility and the enzootic life cycle of *Borrelia burgdorferi*. Infect Immun. 79(8):3273–83. 10.1128/IAI.05153-11 21670168 PMC3147568

[9] He M, Ouyang Z, Troxell B, Xu H, Moh A, Piesman J (2011). Cyclic di-GMP is essential for the survival of the Lyme disease spirochete in ticks. PLoS Pathog. 7(6):e1002133 10.1371/journal.ppat.1002133 21738477 PMC3128128

[10] Caimano MJ, Kenedy MR, Kairu T, Desrosiers DC, Harman M, Dunham-Ems S (2011). The hybrid histidine kinase Hk1 is part of a two-component system that is essential for survival of *Borrelia burgdorferi* in feeding *Ixodes scapularis* ticks. Infect Immun. 79(8):3117–30. 10.1128/IAI.05136-11 21606185 PMC3147546

[11] Kostick JL, Szkotnicki LT, Rogers EA, Bocci P, Raffaelli N, Marconi RT (2011). The diguanylate cyclase, Rrp1, regulates critical steps in the enzootic cycle of the Lyme disease spirochetes. Mol Microbiol. 81(1):219–31. 10.1111/j.1365-2958.2011.07687.x 21542866 PMC3124615

[12] Caimano MJ, Dunham-Ems S, Allard AM, Cassera MB, Kenedy M, Radolf JD (2015). Cyclic di-GMP modulates gene expression in Lyme disease spirochetes at the tick-mammal interface to promote spirochete survival during the blood meal and tick-to-mammal transmission. Infect Immun. 83(8):3043–60. 10.1128/IAI.00315-15 25987708 PMC4496621

[13] Rogers EA, Terekhova D, Zhang H, Hovis KM, Schwartz I, Marconi RT (2009). Rrp1, a cyclic-di-GMP-producing response regulator, is an important regulator of *Borrelia burgdorferi* core cellular functions. Mol Microbiol. 71(6):1551–73. 10.1111/j.1365-2958.2009.06621.x 19210621 PMC2843504

[14] Boardman BK, He M, Ouyang Z, Xu H, Pang X, Yang XF (2008). Essential role of the response regulator Rrp2 in the infectious cycle of *Borrelia burgdorferi*. Infect Immun. 76(9):3844–53. 10.1128/IAI.00467-08 18573895 PMC2519420

[15] Yang XF, Alani SM, Norgard MV (2003). The response regulator Rrp2 is essential for the expression of major membrane lipoproteins in *Borrelia burgdorferi*. Proc Natl Acad Sci U S A. 100(19):11001–6. 10.1073/pnas.1834315100 12949258 PMC196916

[16] Caimano MJ, Groshong AM, Belperron A, Mao J, Hawley KL, Luthra A (2019). The RpoS Gatekeeper in Borrelia burgdorferi: An Invariant Regulatory Scheme That Promotes Spirochete Persistence in Reservoir Hosts and Niche Diversity. Front Microbiol. 10(1923). 10.3389/fmicb.2019.01923 PMC6719511 31507550

[17] Caimano MJ, Eggers CH, Hazlett KR, Radolf JD (2004). RpoS is not central to the general stress response in *Borrelia burgdorferi* but does control expression of one or more essential virulence determinants. Infect Immun. 72(11):6433–45. 10.1128/IAI.72.11.6433-6445.2004 15501774 PMC523033

[18] Hübner A, Yang X, Nolen DM, Popova TG, Cabello FC, Norgard MV (2001). Expression of *Borrelia burgdorferi* OspC and DbpA is controlled by a RpoN-RpoS regulatory pathway. Proc Natl Acad Sci U S A. 98(22):12724–9. 10.1073/pnas.231442498 11675503 PMC60121

[19] Fisher MA, Grimm D, Henion AK, Elias AF, Stewart PE, Rosa PA (2005). *Borrelia burgdorferi* σ^54^ is required for mammalian infection and vector transmission but not for tick colonization. Proc Natl Acad Sci *U S A*. 102(14):5162–7. 10.1073/pnas.0408536102 15743918 PMC555983

[20] Yang XF, Lybecker MC, Pal U, Alani SM, Blevins J, Revel AT (2005). Analysis of the *ospC* regulatory element controlled by the RpoN-RpoS regulatory pathway in *Borrelia burgdorferi*. J Bacteriol. 187(14):4822–9. 10.1128/JB.187.14.4822-4829.2005 15995197 PMC1169512

[21] Caimano M, Iyer R, Eggers C, Gonzalez C, Morton E, Gilbert M (2007). Analysis of the RpoS regulon in *Borrelia burgdorferi* in response to mammalian host signals provides insight into RpoS function during the enzootic cycle. Mol Microbiol. 65(5):1193–217. 10.1111/j.1365-2958.2007.05860.x 17645733 PMC2967192

[22] Ouyang Z, Blevins JS, Norgard MV (2008). Transcriptional interplay among the regulators Rrp2, RpoN, and RpoS in *Borrelia burgdorferi*. Microbiology. 154:2641–58. 10.1099/mic.0.2008/019992-0 18757798

[23] Hyde JA, Shaw DK, Smith Iii R, Trzeciakowski JP, Skare JT (2009). The BosR regulatory protein of *Borrelia burgdorferi* interfaces with the RpoS regulatory pathway and modulates both the oxidative stress response and pathogenic properties of the Lyme disease spirochete. Mol Microbiol. 74(6):1344–55. 10.1111/j.1365-2958.2009.06951.x 19906179 PMC2805275

[24] Ouyang Z, Kumar M, Kariu T, Haq S, Goldberg M, Pal U (2009). BosR (BB0647) governs virulence expression in Borrelia burgdorferi. Mol Microbiol. 74(6):1331–43. 10.1111/j.1365-2958.2009.06945.x 19889086 PMC2831293

[25] Ouyang Z, Deka RK, Norgard MV (2011). BosR (BB0647) controls the RpoN-RpoS regulatory pathway and virulence expression in Borrelia burgdorferi by a novel DNA-binding mechanism. PLoS Pathog. 7(2):e1001272 10.1371/journal.ppat.1001272 21347346 PMC3037356

[26] Dunham-Ems SM, Caimano MJ, Eggers CH, Radolf JD (2012). *Borrelia burgdorferi* requires the alternative sigma factor RpoS for dissemination within the vector during tick-to-mammal transmission. PLoS Pathog. 8(2):e1002532 10.1371/journal.ppat.1002532 22359504 PMC3280991

[27] Miller CL, Karna SLR, Seshu J (2013). *Borrelia* host adaptation regulator (BadR) regulates *rpoS* to modulate host adaptation and virulence factors in *Borrelia burgdorferi*. Mol Microbiol. 88(1):105–24. 10.1111/mmi.12171 23387366 PMC4828661

[28] Ouyang Z, Zhou J. (2015) BadR (BB0693) controls growth phase-dependent induction of *rpoS* and *bosR* in *Borrelia burgdorferi* via recognizing TAAAATAT motifs. Mol Microbiol. 98(6):1147–67. 10.1111/mmi.13206 26331438

[29] Lybecker MC, Samuels DS (2007). Temperature-induced regulation of RpoS by a small RNA in *Borrelia burgdorferi*. Mol Microbiol. 64(4):1075–89. 10.1111/j.1365-2958.2007.05716.x 17501929

[30] Lybecker MC, Abel CA, Feig AL, Samuels DS (2010). Identification and function of the RNA chaperone Hfq in the Lyme disease spirochete *Borrelia burgdorferi*. Mol Microbiol. 78(3):622–35. 10.1111/j.1365-2958.2010.07374.x 20815822 PMC2963666

[31] Kobryn K, Naigamwalla DZ, Chaconas G (2000). Site-specific DNA binding and bending by the Borrelia burgdorferi Hbb protein. Mol Microbiol. 37:145–55. 10.1046/j.1365-2958.2000.01981.x 10931312

[32] Tilly K, Fuhrman J, Campbell J, Samuels DS (1996). Isolation of *Borrelia burgdorferi* genes encoding homologues of DNA-binding protein HU and ribosomal protein S20. Microbiology. 142:2471–9. 10.1099/00221287-142-9-2471 8828214

[33] Arnold WK, Savage CR, Lethbridge KG, Smith TC 2nd, Brissette CA, Seshu J (2018). Transcriptomic insights on the virulence-controlling CsrA, BadR, RpoN, and RpoS regulatory networks in the Lyme disease spirochete. PLoS ONE. 13(8):e0203286–e. 10.1371/journal.pone.0203286 30161198 PMC6117026

[34] Karna SL, Sanjuan E, Esteve-Gassent MD, Miller CL, Maruskova M, Seshu J (2011). CsrA modulates levels of lipoproteins and key regulators of gene expression critical for pathogenic mechanisms of *Borrelia burgdorferi*. Infect Immun. 79(2):732–44. 10.1128/IAI.00882-10 21078860 PMC3028860

[35] Sze CW, Li C (2011). Inactivation of *bb0184*, which encodes carbon storage regulator A, represses the infectivity of *Borrelia burgdorferi*. Infect Immun. 79(3):1270–9. 10.1128/IAI.00871-10 21173314 PMC3067481

[36] Jutras BL, Chenail AM, Carroll DW, Miller MC, Zhu H, Bowman A (2013). Bpur, the Lyme Disease Spirochete's PUR Domain Protein: Identification as a transcriptional modulator and characterization of nucleic acid interactions. J Biol Chem. 288(36):26220–34. 10.1074/jbc.M113.491357 23846702 PMC3764826

[37] Riley SP, Bykowski T, Cooley AE, Burns LH, Babb K, Brissette CA (2009). *Borrelia burgdorferi* EbfC defines a newly-identified, widespread family of bacterial DNA-binding proteins. Nucleic Acids Res. 37(6):1973–83. 10.1093/nar/gkp027 19208644 PMC2665219

[38] Burns LH, Adams CA, Riley SP, Jutras BL, Bowman A, Chenail AM (2010). BpaB, a novel protein encoded by the Lyme disease spirochete's cp32 prophages, binds to erp Operator 2 DNA. Nucleic Acids Res. 38(16):5443–55. 10.1093/nar/gkq284 20421207 PMC2938228

[39] Jutras BL, Chenail AM, Rowland CL, Carroll D, Miller MC, Bykowski T (2013). Eubacterial SpoVG Homologs Constitute a New Family of Site-Specific DNA-Binding Proteins. PLoS ONE. 8(6):e66683 10.1371/journal.pone.0066683 23818957 PMC3688583

[40] Dulebohn DP, Hayes BM, Rosa PA (2014). Global Repression of Host-Associated Genes of the Lyme Disease Spirochete through Post-Transcriptional Modulation of the Alternative Sigma Factor RpoS. PLoS ONE. 9(3):e93141 10.1371/journal.pone.0093141 24671196 PMC3966842

[41] Chen T, Xiang X, Xu H, Zhang X, Zhou B, Yang Y, et al (2018). LtpA, a CdnL-type CarD regulator, is important for the enzootic cycle of the Lyme disease pathogen. Emerg. Microbes Infect. 7(1):126–. 10.1038/s41426-018-0122-1 29985409 PMC6037790

[42] Smith TC, Helm SM, Chen Y, Lin Y-H, Rajasekhar Karna SL, Seshu J (2018). *Borrelia* Host Adaptation Protein (BadP) Is Required for the Colonization of a Mammalian Host by the Agent of Lyme Disease. Infect Immun.86. 10.1128/IAI.00057-18 PMC6013665 29685985

[43] Mason C, Thompson C, Ouyang Z. DksA plays an essential role in regulating the virulence of *Borrelia burgdorferi*. Mol Microbiol. 2020;114(1):172–83. 10.1111/mmi.14504 32227372 PMC8331073

[44] Boyle WK, Groshong AM, Drecktrah D, Boylan JA, Gherardini FC, Blevins JS, et al (2019). DksA controls the response of the Lyme disease spirochete *Borrelia burgdorferi* to starvation. J bacteriol. 201(4):e00582–18. 10.1128/JB.00582-18 30478087 PMC6351744

[45] Tilly K, Krum JG, Bestor A, Jewett MW, Grimm D, Bueschel D (2006). *Borrelia burgdorferi* OspC protein required exclusively in a crucial early stage of mammalian infection. Infect Immun. 74(6):3554–64. 10.1128/IAI.01950-05 16714588 PMC1479285

[46] Liang FT, Jacobs MB, Bowers LC, Philipp MT (2002). An immune evasion mechanism for spirochetal persistence in Lyme borreliosis. J Exp Med. 195:415–22. 10.1084/jem.20011870 11854355 PMC2193615

[47] Liang FT, Yan J, Mbow ML, Sviat SL, Gilmore RD, Mamula M (2004). *Borrelia burgdorferi* changes its surface antigenic expression in response to host immune responses. Infect Immun. 72(10):5759–67. 10.1128/IAI.72.10.5759-5767.2004 15385475 PMC517580

[48] Liang FT, Nelson FK, Fikrig E (2002). Molecular adaptation of *Borrelia burgdorferi* in the murine host. J Exp Med. 196:275–80. 10.1084/jem.20020770 12119353 PMC2193918

[49] Crother TR, Champion CI, Whitelegge JP, Aguiler R, Wu XY, Blanco DR (2004). Temporal analysis of the antigenic composition of *Borrelia burgdorferi* during infection in rabbit skin. Infect Immun. 72(5063):5072. 10.1128/IAI.72.9.5063-5072.2004 PMC517453 15321999

[50] Zhang J-R, Hardham JM, Barbour AG, Norris SJ (1997). Antigenic Variation in Lyme Disease Borreliae by Promiscuous Recombination of VMP-like Sequence Cassettes. Cell. 89(2):275–85. 10.1016/s0092-8674(00)80206-8 9108482

[51] Norris SJ (2014). vls Antigenic Variation Systems of Lyme Disease *Borrelia*: Eluding Host Immunity through both Random, Segmental Gene Conversion and Framework Heterogeneity. Microbiology *spectrum*. 2(6): 10.1128/microbiolspec.MDNA3-0038-2014 26104445 PMC4480602

[52] Chaconas G, Castellanos M, Verhey TB (2020). Changing of the guard: How the Lyme disease spirochete subverts the host immune response. J Bact Chem. 295(2):301–313. 10.1074/jbc.REV119.008583 31753921 PMC6956529

[53] Tilly K, Bestor A, Rosa PA (2013). Lipoprotein succession in Borrelia burgdorferi: similar but distinct roles for OspC and VlsE at different stages of mammalian infection. Mol Microbiol. 89(2):216–27. 10.1111/mmi.12271 23692497 PMC3713631

[54] Labandeira-Rey M, Skare JT (2001). Decreased infectivity in Borrelia burgdorferi strain B31 is associated with loss of linear plasmid 25 or 28–1. Infect Immun. 69:446–55. 10.1128/IAI.69.1.446-455.2001 11119536 PMC97902

[55] Purser JE, Norris SJ (2000). Correlation between plasmid content and infectivity in *Borrelia burgdorferi*. Proc Natl Acad Sci U S A. 97:13865–70. 10.1073/pnas.97.25.13865 11106398 PMC17667

[56] Bankhead T, Chaconas G (2007). The role of VlsE antigenic variation in the Lyme disease spirochete: persistence through a mechanism that differs from other pathogens. Mol Microbiol. 65(6):1547–58. 10.1111/j.1365-2958.2007.05895.x 17714442

[57] Labandeira-Rey M, Seshu J, Skare JT (2003). The Absence of Linear Plasmid 25 or 28–1 of Borrelia burgdorferi Dramatically Alters the Kinetics of Experimental Infection via Distinct Mechanisms. Infect Immun. 71(8):4608–13. 10.1128/iai.71.8.4608-4613.2003 12874340 PMC166013

[58] Fraser CM, Casjens S, Huang WM, Sutton GG, Clayton R, Lathigra R (1997). Genomic sequence of a Lyme disease spirochaete, *Borrelia burgdorferi*. Nature. 390:580–6. 10.1038/37551 9403685

[59] Liang H, Li L, Dong Z, Surette MG, Duan K (2008). The YebC family protein PA0964 negatively regulates the *Pseudomonas aeruginosa* quinolone signal system and pyocyanin production. J Bacteriol. 190(18):6217–27. 10.1128/JB.00428-08 18641136 PMC2546791

[60] Byrne RT, Chen SH, Wood EA, Cabot EL, Cox MM (2014). *Escherichia coli* Genes and Pathways Involved in Surviving Extreme Exposure to Ionizing Radiation. J Bacteriol. 196(20):3534–45. 10.1128/JB.01589-14 25049088 PMC4187691

[61] Brown L, Villegas JM, Elean M, Fadda S, Mozzi F, Saavedra L (2017). YebC, a putative transcriptional factor involved in the regulation of the proteolytic system of *Lactobacillus*. Sci Rep. 7(1):8579 10.1038/s41598-017-09124-1 28819300 PMC5561223

[62] Wei L, Wu Y, Qiao H, Xu W, Zhang Y, Liu X (2018). YebC controls virulence by activating T3SS gene expression in the pathogen *Edwardsiella piscicida*. FEMS Microbiol Lett. 365(14). 10.1093/femsle/fny137 29901702

[63] Kawabata H, Norris SJ, Watanabe H (2004). BBE02 disruption mutants of *Borrelia burgdorferi* B31 have a highly transformable, infectious phenotype. Infect Immun.72(12):7147–54. 10.1128/IAI.72.12.7147-7154.2004 15557639 PMC529111

[64] Indest KJ, Howell JK, Jacobs MB, Scholl-Meeker D, Norris SJ, Philipp MT (2001). Analysis of Borrelia burgdorferi vlsE gene expression and recombination in the tick vector. Infect Immun. 69(11):7083–90. 10.1128/IAI.69.11.7083-7090.2001 11598084 PMC100090

[65] Bykowski T, Babb K, von Lackum K, Riley SP, Norris SJ, Stevenson B (2006). Transcriptional regulation of the *Borrelia burgdorferi* antigenically variable VlsE surface protein. J Bacteriol. 188(13):4879–89. 10.1128/JB.00229-06 16788197 PMC1483003

[66] Drecktrah D, Lybecker M, Popitsch N, Rescheneder P, Hall LS, Samuels DS (2015). The *Borrelia burgdorferi* RelA/SpoT homolog and stringent response regulate survival in the tick vector and global gene expression during starvation. PLoS Pathog. 11(9):e1005160 10.1371/journal.ppat.1005160 26371761 PMC4570706

[67] Hudson CR, Frye JG, Quinn FD, Gherardini FC (2001). Increased expression of *Borrelia burgdorferi vlsE* in response to human endothelial cell membranes. Mol Microbiol 41(1):229–39. 10.1046/j.1365-2958.2001.02511.x 11454215

[68] Brázda V, Coufal J, Liao JCC, Arrowsmith CH (2012). Preferential binding of IFI16 protein to cruciform structure and superhelical DNA. Biochem Biophys Res Commun. 422(4):716–20. 10.1016/j.bbrc.2012.05.065 22618232

[69] Zhang Y, Lin J, Gao Y (2012). In silico identification of a multi-functional regulatory protein involved in Holliday junction resolution in bacteria. BMC Syst Biol. 6 Suppl 1(Suppl 1):S20–S. 10.1186/1752-0509-6-S1-S20 PMC3403352 23046553

[70] Shin DH, Yokota H, Kim R, Kim S-H (2002). Crystal structure of conserved hypothetical protein Aq1575 from *Aquifex aeolicus*. Proc Natl Acad Sci U S A. 99(12):7980–5. 10.1073/pnas.132241399 12060744 PMC123006

[71] Dresser AR, Hardy P-O, Chaconas G (2009). Investigation of the genes involved in antigenic switching at the *vlsE* locus in *Borrelia burgdorferi*: an essential role for the RuvAB branch migrase. PLoS Pathog. 5(12):e1000680–e. 10.1371/journal.ppat.1000680 19997508 PMC2779866

[72] Lin T, Gao L, Edmondson DG, Jacobs MB, Philipp MT, Norris SJ (2009). Central role of the Holliday junction helicase RuvAB in vlsE recombination and infectivity of *Borrelia burgdorferi*. PLoS Pathog. 5(12):e1000679–e. 10.1371/journal.ppat.1000679 19997622 PMC2780311

[73] Brázda V, Laister RC, Jagelská EB, Arrowsmith C (2011). Cruciform structures are a common DNA feature important for regulating biological processes. BMC Mol Biol. 12(1):33. 21816114 10.1186/1471-2199-12-33 PMC3176155

[74] Anguita J, Thomas V, Samanta S, Persinski R, Hernanz C, Barthold SW (2001). *Borrelia burgdorferi*-Induced Inflammation Facilitates Spirochete Adaptation and Variable Major Protein-Like Sequence Locus Recombination. J Immun. 167(6):3383–90. 10.4049/jimmunol.167.6.3383 11544329 PMC4309988

[75] Purser JE, Lawrenz MB, Caimano MJ, Howell JK, Radolf JD, Norris SJ (2003). A plasmid-encoded nicotinamidase (PncA) is essential for infectivity of Borrelia burgdorferi in a mammalian host. Mol Microbiol. 48(3):753–64. 10.1046/j.1365-2958.2003.03452.x 12694619

[76] Lawrenz MB, Wooten RM, Norris SJ (2004). Effects of vlsE Complementation on the Infectivity of Borrelia burgdorferi Lacking the Linear Plasmid lp28-1. Mol Microbiol. 72(11):6577–85. 10.1128/IAI.72.11.6577-6585.2004 PMC523020 15501789

[77] Labandeira-Rey M, Baker EA, Skare JT (2001). VraA (BBI16) protein of *Borrelia burgdorferi* is a surface-exposed antigen with a repetitive motif that confers partial protection against experimental Lyme borreliosis. Mol Microbiol. 69(3):1409–19. 10.1128/IAI.69.3.1409-1419.2001 PMC98035 11179306

[78] Ramsey ME, Hyde JA, Medina-Perez DN, Lin T, Gao L, Lundt ME (2017). A high-throughput genetic screen identifies previously uncharacterized Borrelia burgdorferi genes important for resistance against reactive oxygen and nitrogen species. PLoS Pathog. 13(2):e1006225 10.1371/journal.ppat.1006225 28212410 PMC5333916

[79] Barbour AG (1984). Isolation and cultivation of Lyme disease spirochetes. Yale J Biol Med. 57:521–5. 6393604 PMC2589996

[80] Yang XF, Pal U, Alani SM, Fikrig E, Norgard MV (2004). Essential role for OspA/B in the life cycle of the Lyme disease spirochete. J Exp Med. 199(5):641–8. 10.1084/jem.20031960 14981112 PMC2213294

[81] Samuels DS, Drecktrah D, Hall LS (2018). Genetic transformation and complementation In: Pal U, Buyuktanir O, editors. *Borrelia burgdorferi*: Methods and Protocols. New York, NY: Humana Press p. 183–200. 10.1007/978-1-4939-7383-5_15 PMC5806694 29032546

[82] Bunikis I, Kutschan-Bunikis S, Bonde M, Bergström S (2011). Multiplex PCR as a tool for validating plasmid content of *Borrelia burgdorferi*. J Microbiol Method*s*. 86(2):243–7. 10.1016/j.mimet.2011.05.004 21605603

[83] Elias AF, Bono JL, Kupko JJ, Stewart PE, Krum JG, Rosa PA (2003). New antibiotic resistance cassettes suitable for genetic studies in *Borrelia burgdorferi*. J Mol Microbiol Biotechnol. 6(1):29–40. 10.1159/000073406 14593251

[84] Casjens S, Palmer N, van Vugt R, Huang WM, Stevenson B, Rosa P (2000). A bacterial genome in flux: the twelve linear and nine circular extrachromosomal DNAs in an infectious isolate of the Lyme disease spirochete *Borrelia burgdorferi*. Mol Microbiol. 35:490–516. 10.1046/j.1365-2958.2000.01698.x 10672174

