# Supplementary material for: YebC regulates variable surface antigen VlsE expression and is required for host immune evasion in Borrelia burgdorferi
Source: PLoS Pathog. 2020 Oct 13;16(10):e1008953. doi: 10.1371/journal.ppat.1008953 (PMC7584230; doi:10.1371/journal.ppat.1008953)
Supplement: S1 Table — (DOCX) [file ppat.1008953.s001.docx]

**Supplemental Table S1. Strains, plasmids, and primers used in this study.**

|  | **Description or sequence** | **Source or purpose** |
| --- | --- | --- |
| **Strains** |  |  |
| 5A4NP1 | Wild-type strain with *bbe02* gene disrupted by Kan^r^ | [[57](#_ENREF_57)] |
| ΔyebC | *yebC* deletion mutant; 5A4NP1 transformed with pCT007 | This study |
| *yebC*^com^ | Complemented *yebC* mutant ; Δ*yebC* transformed with pCT016 | This study |
| **Plasmids** |  |  |
| pCT007 | Suicide vector for constructing *bb0025* deletion; Str^r^ | This study |
| pCT016 | pBSVG Shuttle vector carrying *yebC* gene with its native promoter; Gen^r^ | This study |
| **Primers** |  |  |
| PRCT017 | ACGGGGCCCTAGCAAGGCAAAATGCACTAACT | Construct pCT007 |
| PRCT018 | ACGGTCGACTAATTCTTCTTGTTGAAAAGCTTGAAGA | Construct pCT007 |
| PRCT019 | TCCCCCCGGGTTACACCTCCAACAATTTTTCCA | Construct pCT007 |
| PRCT020 | GCTCTAGATATAATTTATTGAAAATGTAATAGGCACGC | Construct pCT007 |
| PRCT092 | AAAACTGCAGCTAACTTAATTCTTCTGGGA | Construct pCT016 |
| PRCT096 | CGCGGATCCGGCCGCAATTTTTTTTATGTTTACACCA | Construct pCT016 |
| PRCT021-P1 | CTACTCCGCTTACTCCAATAAGCT | *yebC* mutant verification |
| PRCT022-P2 | AGTTCTTAAATTTGTAATATTGTTGTTTCTATCT | *yebC* mutant verification |
| YZ-aadA-5-P3 | AAGTATCGACTCAACTATCAGAGGT | *yebC* mutant verification |
| YZ-aadA-3-P4 | CCATAGCGTTAAGGTTTCATTTAGC | *yebC* mutant verification |
| PRCT023-P5 | AGGAATTAAAGCTATCTCTGCCAC | *yebC* mutant verification |
| PRCT024-P6 | GTGCTCTTGATGCAAAGCGTAAT | *yebC* mutant verification |
| BB0025 RT PCR-F | ATGTCTGGTCACAGTAAATGGTCAA | qRT-PCR for *yebC* |
| BB0025 RT PCR-R | AACCTTAGCCTTATTAACAGCTACC | qRT-PCR for *yebC* |
| YZ-qPCR-flaB-F2 | CACCAGCATCACTTTCAGGGTCTCA | qRT-PCR for *flaB* |
| YZ-qPCR-flaB-R2 | TGTAGCAGGTGCTGGCTGTTGA | qRT-PCR for *flaB* |
| qPCR-VlsE-F | CTGAGTCTGCAGTTCGCAAAGTT | qRT-PCR for *vlsE* |
| qPCR-VlsE-R | TCACTGAATCACCGACTTTCCTTA | qRT-PCR for *vlsE* |
| qPCR *rpoS* FP | TGCAGGACAAATACAAAGAGGC | qRT-PCR for *rpoS* |
| qPCR *rpoS* RP | TCGGGTCATATTTTTCAGCAGC | qRT-PCR for *rpoS* |
| qPCR *bbi16* FP | CTGTTTTAAGCCCCCTGGAT | qRT-PCR for *bbi16* |
| qPCR *bbi16* RP | CTTTTCCTGCAACACTTCCTTT | qRT-PCR for *bbi16* |
| qPCR *bba49* FP | ATGAATAAGCAAGAGATTGCGAC | qRT-PCR for *baA49* |
| qPCR *bba49* RP | CGGTTAACCTCTACAAGCTCC | qRT-PCR for *bba49* |
| qPCR *bba51* FP | TCAGCCCGTGCAGTAATTG | qRT-PCR for *bba51* |
| qPCR *bba51* RP | ACATGCTGTAATAAACCCCCA | qRT-PCR for *bba51* |
| bosR RP | TTCATTCCTAATTCTGATG | PCR for EMSA probe |
| bosR 350 UP FP | CATATCATTCATACAATTAATTA | PCR for EMSA probe |
| *vlsE* 200 bp EMSA FP | CTTCCGTATCACATATAATAAAGT | PCR for EMSA probe |
| *vlsE*  EMSA RP | GTTGTTAATAAAATTGCACTTG | PCR for EMSA probe |
